# Enhanced Genome Editing Activity with Novel Chimeric ScCas9 Variants in Rice

**DOI:** 10.1002/advs.202411549

**Published:** 2025-01-04

**Authors:** Zhen Liang, Yuqing Wu, Shuke Deng, Sha Wei, Kai Zhang, Yingjie Guo

**Affiliations:** ^1^ School of Life Science Shanxi University Taiyuan Shanxi 030006 China; ^2^ Research Institute of Big Data Science and Industry Shanxi University Taiyuan Shanxi 030006 China

**Keywords:** genome editing, plants, protein engineering, Sc++, SpcRN++

## Abstract

The *Streptococcus canis* Cas9 protein (ScCas9) recognizes the NNG protospacer adjacent motif (PAM), offering a wider range of targets than that offered by the commonly used *S. pyogenes* Cas9 protein (SpCas9). However, both ScCas9 and its evolved Sc++ variant still exhibit low genome editing efficiency in plants, particularly at the less preferred NTG and NCG PAM targets. In this study, a chimeric SpcRN++ variant is engineered by grafting the recognition (REC) domain of SpCas9 into the Sc++ variant, incorporating the R221K/N394K mutations, and retaining the positively charged loop of *S. anginosus* Cas9. The SpcRN++ variant exhibits a higher genome editing capacity and wider target range than the Sc++ variant in rice protoplasts and stable transgenic plants. Further evidence indicates that nSpcRN++‐based A3A/Y130F and TadA8e exhibit enhanced cytosine and adenine editing efficiency in plants. Finally, herbicide‐resistant rice germplasms are produced by targeting the *OsACC* gene using nSpcRN++‐based adenine base editors. These results demonstrate that SpcRN++ is a powerful tool for genome editing in plants, and this integrative protein engineering strategy holds promise for engineering other Cas9 proteins.

## Graphical abstract

This study engineers a chimeric SpcRN++ variant by grafting the recognition (REC) domain of SpCas9 into the Sc++ variant, incorporating the evolutionary R221K/N394K mutations, and retaining the positively charged loop from *S. anginosus* Cas9. SpcRN++ demonstrates superior genome editing capability than Sc++ in rice. SpcRN++ also exhibits high compatibility with base editing tools, making it suitable for trait improvement applications.

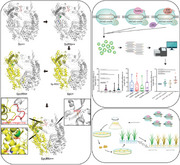

## 1 Introduction

Over the past decade, CRISPR/Cas9 genome editing technology has rapidly advanced animal and plant breeding, as well as gene therapy, due to its simplicity and high efficiency.^[^ ^1^, ^2^ ^]^ The CRISPR/Cas9 system searches for potential target sites in a genome by recognizing the presence of protospacer adjacent motif (PAM) sequences, and then induces DNA double‐strand breaks. For instance, the commonly used SpCas9 from *Streptococcus pyogenes* recognizes canonical NGG PAM, which significantly restricts its range of targets.^[^ ^3^, ^4^ ^]^ Base editing technologies derived from the CRISPR/Cas9 system, such as cytosine base editors (CBEs),^[^ ^5^ ^]^ adenine base editors (ABEs),^[^ ^6^ ^]^ and glycosylase‐based base editors (GBEs),^[^ ^7^ ^]^ can create gain‐of‐function mutations at specific bases of the target genes. However, their editing window is constrained by the requirement for a PAM sequence at the target site.^[^ ^8^ ^]^ Prime editors can theoretically enable precise base substitutions and facilitate small fragment DNA insertion/deletion mutations. However, their efficiency depends on the distance between the editing site and the PAM sequence site.^[^ ^9^, ^10^ ^]^ Therefore, the selection and flexibility of PAM sequence sites are crucial for precise gene editing.

To expand the range of gene editing targets, researchers have long been committed to engineering the Cas9 protein to enhance its compatibility with PAM sequences.^[^ ^11^ ^]^ Notably, the development of xCas9, Cas9‐NG, and SpG variants that recognize NG PAM sequences and the SpRY variant that recognizes NNN (NRN>NYN) PAM sequences has significantly improved the accessibility of genome editing tools.^[^ ^12^, ^13^, ^14^ ^]^ However, expansion of the range of PAM sites recognized by these four Cas9 variants has occurred at the expense of their editing activity.^[^ ^15^, ^16^, ^17^, ^18^ ^]^ In particular, the SpRY variant displays restricted editing efficiency, pronounced self‐editing, and off‐target effects.^[^ ^19^, ^20^ ^]^

Bioinformatics‐based mining of Cas9 proteins can also be used to enrich the PAM sequences. Notably, ScCas9 from *S. canis*, which has NNG PAM recognition ability, is the only naturally occurring Cas nuclease that recognizes a single‐base PAM sequence.^[^ ^21^ ^]^ ScCas9 was further upgraded through bioinformatics analysis and structure‐guided design, incorporating the T1227K mutation from *S. gordonii* Cas9 and a positively charged loop (positions 367–376) from *S. anginosus* Cas9, resulting in an Sc++ variant with significantly enhanced editing activity. The Sc++ variant exhibited superior editing efficiency compared with xCas9 and SpCas9‐NG in mammalian cells.^[^ ^22^ ^]^ In plants, the Sc++ variant also shows higher editing efficiency than ScCas9.^[^ ^23^, ^24^ ^]^ Sc++ demonstrates strong cutting ability at the NAG and NGG PAM targets; however, its efficiency at the NCG and NTG PAM targets is limited. Some research groups have suggested that the Sc++ variant recognizes NVG PAM sequences (V = A, G, or C).^[^ ^23^ ^]^ These findings suggest that while the Sc++ variant displays superior editing capabilities compared to other Cas nucleases and their variants that recognize single‐base PAMs, its editing efficiency requires further optimization.

Optimizing the performance of current genome editing tools requires more sophisticated protein engineering efforts than previously undertaken. The high‐resolution structures of Cas9 and Cas12a nucleases provide valuable insights into rational protein design strategies. Various research groups have endeavored to enhance the genome editing capabilities of Cas9 proteins through rational mutagenesis of their target DNA‐binding amino acids.^[^ ^13^, ^14^, ^25^, ^26^ ^]^ Cas9 variants engineered by replacing non‐ or negatively charged residues in the nuclease (RuvC or HNH) domain with positively charged arginine residues have also been used to increase affinity for the DNA substrate, thereby improving the editing efficiency.^[^ ^27^ ^]^ Additionally, directed evolution and site‐saturated mutagenesis have been shown to be effective in altering the characteristics of target proteins without the need for detailed structural or mechanistic information. These approaches have been utilized to increase the nuclease activity and target scope of both Cas9 and the less active Cas12 proteins.^[^ ^28^, ^29^, ^30^, ^31^ ^]^ Furthermore, sequence conservation of Cas9 orthologs mapped onto its structure suggests domains that may be malleable for engineering. Phylogenetic analysis revealed high sequence diversity in the PAM‐interacting (PI) domain among Cas9 orthologs. Swapping the PI domain between Cas9 orthologs creates several chimeric Cas9 variants with expanded PAM compatibility.^[^ ^32^, ^33^, ^34^ ^]^ The recognition (REC) domain of Cas9 plays a crucial role in target DNA cleavage. However, its potential role in enhancing genome editing efficiency through synthetic chimeric nucleases has been overlooked.

In this study, we engineered chimeric Cas9 variants by combining Sc++ and SpCas9 to enhance the genome editing capability of ScCas9. To achieve this, we initially attempted to graft the REC domain of SpCas9 into the Sc++ enzyme. However, the resulting chimeric enzyme exhibited reduced editing efficiency in rice plant cells. We then stacked two point mutations (R221K and N394K) and a positively charged loop (positions 367–376) from *S. anginosus* Cas9 to create a chimeric Sp‐RN‐loop‐Sc++ (herein referred to as SpcRN++). The SpcRN++ variant exhibited enhanced nuclease activity compared with Sc++ at diverse NNG PAM targets in rice protoplast cells and transgenic plants. Even the target sites that were resistant to Sc++ were successfully edited using the SpcRN++ variant. We further showed that nSpcRN++ fused with a cytosine or adenine deaminase achieved the highest base editing efficiency among all variants in rice. We also used nSpcRN++‐based adenine base editors to generate herbicide‐resistant rice. In summary, we present SpcRN++ as a novel chimeric ScCas9 variant with augmented genome editing capabilities for NNG PAM targets in plants. This engineered Sc++‐based chimeric variant is expected to facilitate robust genome editing and expand its possible applications in plant biology.

## 2 Results

### 2.1 Engineering of Chimeric ScCas9 Variants with Enhanced Activity

**Figure 1:**
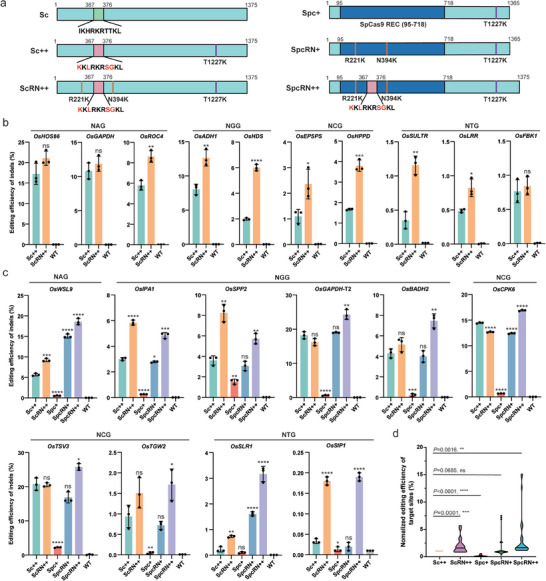
Design and characterization of DNA cleavage activities of Sc++‐ based variants in plant cells. a) Schematic illustration of ScCas9 and its derivatives, Sc++, ScRN++, Spc+, SpcRN+, and SpcRN++. The green box in Sc indicates amino acid sequences from 367 to 376 of the ScCas9 protein. The pink box in Sc++, ScRN++, and SpcRN++ represents the positively charged loop (positions 367–376) of *S. anginosus* Cas9. The dark blue box in Spc+, SpcRN+, and SpcRN++ represents the REC domain (positions 95–718) of SpCas9. b) Evaluation of editing efficiency of Sc++ and ScRN++ at 10 target sites with NNG PAMs in rice protoplasts. c) Comparison of the editing efficiency achieved by chimeric nucleases Spc+, SpcRN+, and SpcRN++ nucleases with that of Sc++ and ScRN++ at an additional 10 target sites with NNG PAMs in rice protoplasts. Editing efficiencies (mean ± s.e.m.) are based on three independent experiments (*n* = 3) in (b) and (c). d) Overall editing efficiencies achieved by Sc++, ScRN++, Spc+, SpcRN+, and SpcRN++. The editing efficiencies achieved by Sc++ for each target were normalized to 1, and the efficiencies of ScRN++, Spc+, SpcRN+, and SpcRN++ for each target were adjusted accordingly. *p‐values* were determined using the two‐tailed Student's *t*‐test: ^*^ *p* < 0.05, ^**^ *p* < 0.01.

ScCas9 shares 89.2% sequence similarity with the commonly used and highly efficient SpCas9.^[^ ^21^ ^]^ To enhance the nuclease activity of ScCas9, we initially attempted to mimic the R221K/N394K mutations, which have been shown to increase the editing efficiency of SpCas9 based on sequence alignment.^[^ ^31^ ^]^ We commercially synthesized a plant codon‐optimized Sc++ variant and introduced the R221K/N394K mutation into its coding region to generate an ScRN++ variant (**Figure** **1**a; Sequences, Supporting Information). To investigate their nuclease activity and PAM specificity, we designed 10 single guide RNAs (sgRNAs) that targeted the rice endogenous genomic loci with NAG, NGG, NCG, and NTG PAMs. SgRNAs under the control of the rice U3 (*OsU3*) promoter, along with the nuclear localization signal (NLS)‐flanked Sc++ and its variant driven by the maize Ubiquitin1 (*ZmUbi*) promoter, were delivered into rice protoplasts (Figure S1a, Supporting Information). Two days after transfection, the genomic DNA was extracted and subjected to amplicon deep sequencing. Sequencing analysis showed that ScRN++ exhibited a higher editing efficiency than Sc++ at seven of the ten target sites, whereas the editing efficiency was comparable at the remaining three target sites (Figure 1b). For the NAG and NGG PAMs, the editing efficiency of Sc++ ranged from 2.0% to 17.2%, whereas that of ScRN++ was, on average, 1.7‐fold higher at 6.0% to 21.1% (except for the *OsHOS66* and *OsGAPDH* targets). For the NCG and NTG PAMs, Sc++ yielded an editing efficiency of 0.4–1.7%, and that of ScRN++ was, on average, 2.5‐fold higher at 0.8–3.8% (except for the *OsFBK1* target). The observed variations in editing efficiency across different PAM targets were also consistent with the findings of previous studies that tested ScCas9 and Sc++ in rice and demonstrated that their editing efficiency at the NAG and NGG PAM targets was superior to that at the NTG and NCG PAM targets.^[^ ^23^, ^24^, ^35^, ^36^ ^]^ Taken together, these results demonstrate that the R221K/N394K mutations in ScRN++ moderately enhance its nuclease activity.

Considering the modular structure of Cas9 enzymes, we postulated that engineering chimeric Cas9 variants would generate non‐natural variants that would expand the kinetic parameter spectrum.^[^ ^37^ ^]^ The Cas9 protein comprises a REC lobe responsible for nucleic acid binding and activation of the HNH domain.^[^ ^38^ ^]^ Notably, the R221K/N394K mutations in the REC domain have been shown to enhance the nuclease activity of both SpCas9 and Sc++ (Figure 1b), thereby underscoring the pivotal roles of the REC domain in DNA cleavage.^[^ ^31^ ^]^ Moreover, SpCas9 demonstrated higher editing efficiency than Sc++ at the NGG PAM sites.^[^ ^35^ ^]^ Inspired by these findings, we sought to engineer variants that would embody the salient features of Sc++ and SpCas9. To this end, we rationally replaced the REC domain of Sc++ with that of SpCas9, resulting in a chimeric hybrid Cas9, which we termed Spc+ (Figure 1a). Furthermore, the R221K/N394K mutations were incorporated into Spc+, either independently or in conjunction with the positively charged loop (positions 367–376) of *S. anginosus* Cas9, to yield SpcRN+ and SpcRN++ variants, respectively (Figure 1a; Sequences, Supporting Information). To evaluate the on‐target efficacy of these engineered chimeric Cas9 variants, we selected ten additional target sites across the NAG, NGG, NCG, and NTG PAMs (Table S1, Supporting Information) and assessed the editing efficiency using a transient protoplast assay. Sc++ and ScRN++ were used as benchmark controls. Notwithstanding the observation that the Spc+ variant exhibited markedly diminished activity compared to Sc++, incorporation of the R221K/N394K mutations in Spc+, which yielded SpcRN+, successfully restored its cleavage activity and achieved an editing efficiency comparable to that of Sc++ (Figure 1c). Notably, the chimeric SpcRN++ variant, which features the SpCas9 REC lobe, the R221K/N394K mutations, and the positively charged loop of *S. anginosus* Cas9, exhibited the best performance among all the variants (Figure 1d; Figure S2, Supporting Information). SpcRN++ consistently exhibited superior editing efficiency compared to Sc++ across all ten tested targets, and ScRN++ showed enhanced efficiency at five of these targets (Figure 1c). Moreover, SpcRN++ exhibited a higher editing efficiency than ScRN++ at eight of these targets. In terms of on‐target efficiency, SpcRN++ was, on average, 3.3‐fold higher (maximum 14.2‐fold for *OsSLR1*) than Sc++. In comparison, ScRN++ demonstrated an average increase of approximately 2.0‐fold in on‐target efficiency (maximum 5.4‐fold for *OsSIP1*) relative to Sc++ (Figure 1c,d). The greatest enhancements were observed for both SpcRN++ and ScRN++ at the NTG PAM targets, which were previously reported to be resistant to Sc++.^[^ ^23^ ^]^ Consequently, we concluded that relative to Sc++, both SpcRN++ and ScRN++ variants had enhanced editing efficiency, with SpcRN++ offering slightly higher efficiency than ScRN++ in plant cells.

### 2.2 Chimeric SpcRN++ Outperformed Other Variants in Stable Transgenic Rice

We further conducted a comprehensive evaluation of the editing performance of the engineered ScCas9 variants in transformed plants. We selected 12 targets in the rice endogenous genomic loci, five of which were previously assessed in the protoplast assay (Table S1, Supporting Information). Given the suboptimal editing efficiency observed for the NCG and NTG PAM targets, we selected two targets each for the NAG and NGG PAMs and four each for the NCG and NTG PAMs. Subsequently, we constructed six multiplex genome editing vectors for the 12 targets in rice (Figure S1b, Supporting Information). Multiplex targeting vectors incorporating Sc++, ScRN++, SpcRN+, and SpcRN++ were independently delivered into rice plants via *Agrobacterium*‐mediated stable transformation. Typically, 30 to 40 transgenic plants were generated for each vector and the mutations were detected using the High‐throughput Tracking of Mutations (Hi‐TOM) platform.^[^ ^39^ ^]^

**Figure 2:**
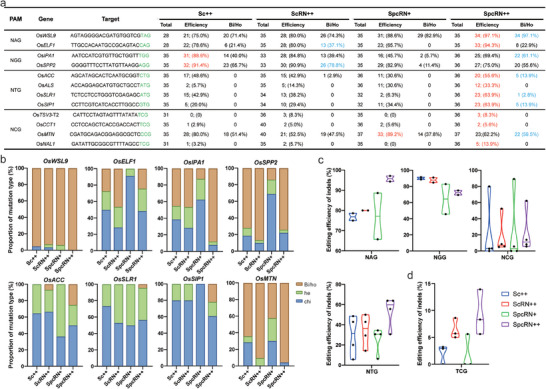
Robust genome editing by SpcRN++ in transgenic rice plants. a) Summary of the editing efficiencies and bi‐allelic ratios induced by Sc++, ScRN++, SpcRN+, and SpcRN++ at 12 target sites with NNG PAMs in T_0_ transgenic plants. The highest editing efficiencies and bi‐allelic ratios are highlighted in red and blue, respectively. b) Analysis of various mutation types induced by Sc++, ScRN++, SpcRN+, and SpcRN++ at eight target sites harboring homozygous or bi‐allelic mutants. Bi, bi‐allelic; Ho, homozygous; He, heterozygous; chi, chimeric. Seedlings with >70% indel frequencies were classified as bi‐allelic or homozygous mutations, those with 30% to 70% indel frequencies were considered heterozygous mutations, and those with <30% indel frequencies were deemed chimeric mutations. c) Editing efficiencies at NAG, NGG, NTG, NCG, and TCG PAM targets in the T_0_ plants.

Sequencing analysis indicated that SpcRN++ exhibited the highest genome editing efficiency among all variants at the NAG PAM targets, with 97.1% at *OsWSL9* and 94.3% at *OsEFL1* (**Figure** **2**a). However, for the NGG PAM targets, all variants yielded lower editing efficiencies than Sc++. Nevertheless, SpcRN++ demonstrated a comparable or higher bi‐allelic mutation ratio than Sc++. For the *OsIPA1* targets, 88% (22/25) of the mutants generated by SpcRN++ were homozygous/bi‐allelic, whereas the corresponding percentages were only 45.2%, 46.4%, and 12.5% for Sc++, ScRN++, and SpcRN+, respectively (Figure 2a,b). Comparable homozygous/bi‐allelic ratios were achieved for the *OsSPP2* target using Sc++, ScRN++, and SpcRN++. For the NTG PAM targets, SpcRN++ exhibited the highest on‐target efficiency among all four variants for all the tested targets (Figure 2c). The editing efficiency of SpcRN++ ranged from 33.3% to 63.9% at these four targets, while those of Sc++, ScRN++, and SpcRN+ were 5.7~48.6%, 14.3~42.9%, and 6.3~34.4%, respectively. Furthermore, the bi‐allelic mutants were exclusively present at the SpcRN++‐targeted sites, with the exception of one bi‐allelic mutant generated by ScRN++ at the *OsACC* target (Figure 2a). The on‐target effects of Sc++ and its variants varied in the NCG PAM targets. The *OsMTN* target was efficiently edited by all variants, with SpcRN+ showing the highest editing efficiency and SpcRN++ yielding the highest bi‐allelic mutation ratio (Figure 2a,b). However, Sc++ and SpcRN+ exhibited negligible activity against the remaining three targets (*OsTSV3‐T2*, *OsCCT1*, and *OsNAL1*), all of that contained TCG PAMs. Intriguingly, SpcRN++ successfully targeted the three targets with an editing efficiency of 5.6~13.9%, whereas the editing efficiency of ScRN++ was 5.0~8.3% (Figure 2a,c; Figure S3, Supporting Information). No significant differences were observed between the mutation types produced by the four variants (Figures S4 and S5, Supporting Information). These results indicate that SpcRN++ is a promising chimeric Cas9 nuclease for generating genome‐edited rice plants with NNG PAM targets owing to its enhanced editing activity.

### 2.3 SpcRN++ Exhibits Low Off‐Target Activity During Genome Editing in Plants

**Figure 3:**
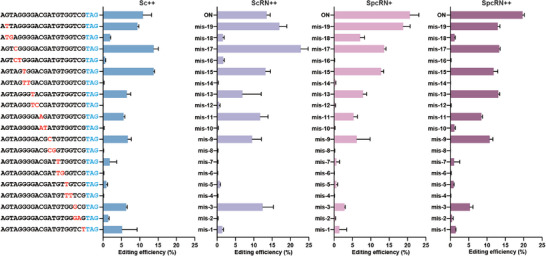
Comparison of sequence specificities of Sc++, ScRN++, SpcRN+, and SpcRN++. Off‐target effects of Sc++ and its variants ScRN++, SpcRN+, and SpcRN++ with guide sequences containing singular or double mismatches at successive positions against the *OsWSL9* target. Each sgRNA was tested in combination with the four ScCas9 variants, and the percentage of indels was used to measure the editing activity. Editing efficiencies (mean ± s.e.m.) are based on three independent experiments (*n* = 3). The WT guide sequence is highlighted in bold, and the PAM is highlighted in blue. The mismatch sites are highlighted in red.

The potential for off‐target effects remains a significant concern for CRISPR/Cas9‐mediated genome editing applications. Previous studies have demonstrated that Sc++ is an inherently high‐fidelity nuclease that exhibits fewer off‐target effects than SpCas9.^[^ ^22^ ^]^ We further characterized the editing fidelity of the engineered chimeric Cas9 variants using a mismatch tolerance assay. We systematically introduced single or adjacent double mismatches at consecutive positions along the guide sequence of the *OsWSL9* and *OsSLR1* targets (Table S2, Supporting Information). Subsequently, we quantified the genome editing efficiency of mismatched sgRNAs against the on‐target sequence. We found that all variants had a higher tolerance to single mismatches than to double mismatches (**Figure** **3**; Figure S6, Supporting Information). Notably, the SpcRN++ variant exhibited slightly diminished sequence specificity for some single mismatches compared with Sc++. This may be attributed to a trade‐off for its enhanced activity (Figure 3).

To further investigate the potential off‐target effects in transgenic plants, we employed the CRISPR‐GE online tool to predict candidate off‐target sites with up to three mismatches against three on‐target sites (*OsIPA1*, *OsWSL9*, and *OsSPP2*) (Table S3, Supporting Information).^[^ ^40^ ^]^ A total of 12 bi‐allelic edited plants were selected for each treatment, and the potential off‐target mutations were detected using Hi‐TOM sequencing. The only exceptions were the *OsIPA1* and *OsSPP2* targets edited by SpcRN+, which had only two and four bi‐allelic edited plants in the T_0_ generation, respectively (Figure S7, Supporting Information). The sequencing results demonstrated that none of the Sc++, ScRN++, SpcRN+, and SpcRN++ nucleases exhibited any off‐target effects at the targets with two or three mismatches. Therefore, we considered that SpcRN++ largely maintained the intrinsic sequence specificity of Sc++, while offering enhanced activity.

### 2.4 Evaluation of Chimeric Cas9 Variants‐Based Base Editing in Rice Cells

**Figure 4:**
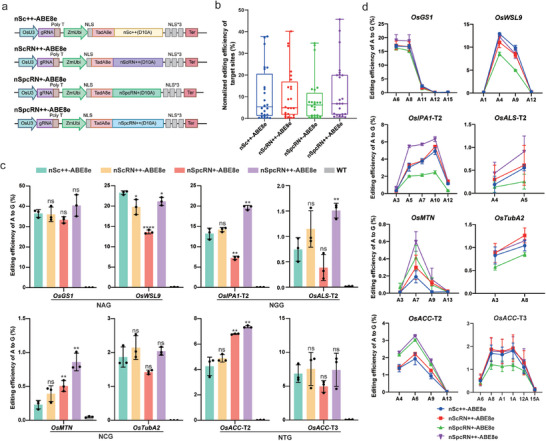
Comparative analysis of adenine base editing efficiency achieved by Sc++‐ based variants in rice protoplasts. a) Schematic illustration of ABE constructs with TadA8e‐fused nSc++, nScRN++, nSpcRN+ and nSpcRN++. b) Overall editing efficiencies at eight target sites induced by nSc++‐, nScRN++‐, nSpcRN+‐ and nSpcRN++‐based ABEs. c) Efficiencies of targeted A‐to‐G conversion at eight targets with NNG PAMs achieved by the four ScCas9‐derived variants. These efficiencies were calculated by summing the A‐to‐G conversions for all sites within the editing window. *p‐values* were obtained using two‐tailed Student's *t*‐test: ^*^ *p* < 0.05, ^**^ *p* < 0.01. d) Adenine editing window of the eight target sites induced by the four designed ABE8e vectors. Editing efficiencies (mean ± s.e.m.) are based on three independent experiments (*n* = 3).

The PAM preferences of Cas proteins significantly constrain the target range of base editors. Considering the singular base PAM requirement demonstrated by Sc++ and its derivatives, we investigated their adenine base editing efficiency and compatibility. The fusion constructs were engineered by linking hyperactive adenosine deaminase TadA8e to the amino termini of the D10A nickase variants, generating nSc++‐, nScRN++‐, nSpcRN+‐, and nSpcRN++‐based adenine base editors (ABEs) (**Figure** **4**a). Subsequently, we selected eight target sites in rice endogenous loci with NAG, NGG, NCG, and NTG PAMs and measured their A‐to‐G efficiency in rice protoplasts using amplicon deep sequencing. The results demonstrated that the overall base editing efficiencies of nScRN++‐, nSpcRN+‐, and nSpcRN++‐based ABE8e were comparable to those of nSc++ (Figure 4b). Notably, for targets exhibiting relatively low editing efficiency (<10% A‐to‐G conversion), nSpcRN++‐based ABE8e increased the base editing ratio, achieving a maximum enhancement of 3.4‐fold at the *OsMTN* target (Figure 4c). Conversely, at other targets where the nSc++‐based ABE8e was already effective, nSpcRN++ demonstrated a limited enhancement of the base editing ratio, with only a 1.4‐fold improvement observed at the *OsIPA1*‐T2 target (Figure 4c). Further analysis of the base editing profiles across the eight targets at the editable adenine nucleotides revealed that the editing window (positions 4–10) for all variants nearly overlapped, with limited effects outside the main editing window (Figure 4d). In summary, these results indicate that SpcRN++ likely exhibits enhanced compatibility with TadA8e at loci challenged by suboptimal efficiency.

**Figure 5:**
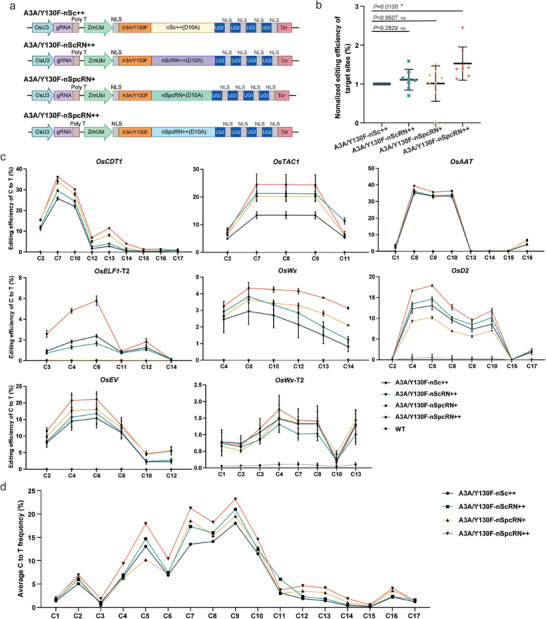
Comparison of the C‐to‐T base editing efficiency of Sc++ variants‐ based CBE in rice protoplasts. a) Schematic representation of CBE constructs with A3A/Y130F‐fused nSc++, nScRN++, nSpcRN+, and nSpcRN++. b) Overall comparison of the base editing efficiency of cytosine base editors derived from four ScCas9 variants. Editing efficiencies represent only the highest edited cytosine of each target. Editing efficiencies achieved by A3A/Y130F‐nSc++ for each target were normalized to 1, and the efficiencies of nScRN++, nSpcRN+, and nSpcRN++‐based CBEs for each target were adjusted accordingly. *p‐values* were obtained using two‐tailed Student's *t*‐test: ^*^ *p* < 0.05. c) The C‐to‐T conversion efficiency of nSc++‐, nScRN++‐, nSpcRN+‐, and nSpcRN++‐based CBEs at eight endogenous genomic loci in rice cells. d) Average C‐to‐T base editing efficiency at different positions in eight targets edited by the four ScCas9‐derived variants. Editing efficiencies (mean ± s.e.m.) are based on three independent experiments (*n* = 3).

Based on these results, we sought to use SpcRN++ for C‐to‐T base editing in plants. Cytosine base editors were generated by fusing the D10A nickase of the four ScCas9 variants with A3A/Y130F, an APOBEC3A (A3A) cytosine deaminase variant that incorporates the Y130F mutation (**Figure** **5**a).^[^ ^41^ ^]^ Subsequently, we evaluated the efficacy of these base editors at eight endogenous loci in rice protoplasts. Amplicon deep sequencing data revealed that A3A‐nSpcRN++ displayed the most robust C‐to‐T conversion efficiency across all target sites, with an average 1.5‐fold enhancement in efficiency relative to A3A/Y130F‐nSc++. In contrast, the base editing efficiencies of A3A/Y130F‐nScRN++ and A3A/Y130F‐nSpcRN+ were comparable to those of A3A/Y130F‐nSc++ (Figure 5b; Figure S8, Supporting Information). Further analysis was conducted to determine whether the editing window of the CBE system was altered when the ScCas9 variants were used. Among the eight tested targets, four CBEs exhibited consistent editing windows of C3‐C11 (Figure 5c,d), and other Cs outside the main editing window displayed lower editing efficiency. These results indicate that the SpcRN++ variant exhibited higher compatibility with A3A/Y130F deaminase, suggesting that the fusion of other cytosine deaminases could further enrich the base editing toolbox.

### 2.5 nSpcRN++‐Based ABE Outperformed Other Variants in Transgenic Plants

**Figure 6:**
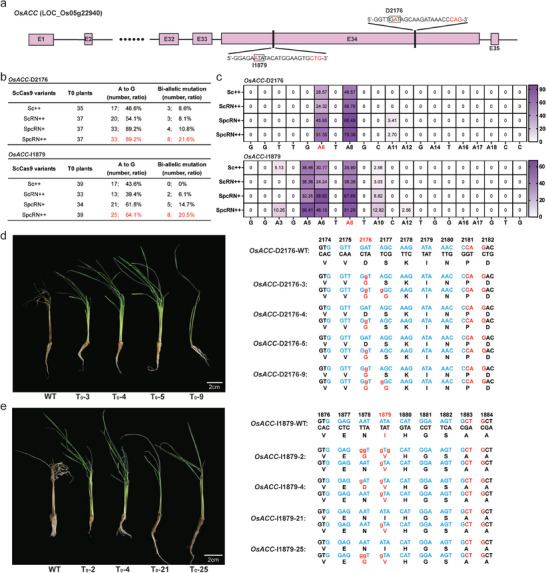
Robust base editing activity of nSpcRN++‐TadA8e in transgenic plants and herbicide resistance conferred to rice. a) Schematic representation of the target sites in the *OsACC* gene. b) Editing efficiencies and bi‐allelic ratio induced by nSc++‐, nScRN++‐, nSpcRN+‐, and nSpcRN++‐based ABEs at *OsACC*‐D2176 and ‐I1879 targets in T_0_ transgenic plants. c) Heat maps illustrating the efficiencies of targeted single A‐to‐G conversion and editing window at the *OsACC*‐D2176 and ‐I1879 targets induced by the four ABE8es. The A‐to‐G substitutions in *OsACC*‐D2176 (A6) and *OsACC*‐D1879 (A8) that may confer herbicide resistance were highlighted in red. Herbicide‐resistant T_0_ lines were generated by targeting amino acids D2176 d) and I1879 e) and their genotypes. Scale bars: 2 cm.

We further evaluated the performance of our designed chimeric Cas9 variants in mediating adenine base editing in transgenic plants and explored their potential applications in agricultural trait improvement. To elucidate these potential applications, we used Sc++‐based variants for adenine base editing to engineer herbicide‐resistant rice by targeting the *OsACC* gene. We designed two sgRNAs targeting the DNA sequences encoding the pivotal amino acids D2176 and I1879 in the carboxyl‐transferase (CT) domain of *OsACC*. Analogous substitutions of these two amino acids have previously been demonstrated to confer herbicide resistance in *L. rigidum*.^[^ ^42^ ^]^ One of the sgRNAs recognized the NAG PAM, and the other specifically recognized the NTG PAM (**Figure** **6**a). The two sgRNAs were subsequently incorporated into the ABE8e vectors based on nSc++, nScRN++, nSpcRN+, and nSpcRN++ (Figure 4a). The eight resulting constructs were used for rice transformation. Approximately 40 transgenic plants of each construct were regenerated and genotyped using Hi‐TOM sequencing.

For the *OsACC*‐D2176 target, 33 of the 37 nSpcRN++‐ABE8e transformed lines harbored at least one A‐to‐G conversion in the target region, resulting in a mutation efficiency of 89.2%. This efficiency was comparable to that of nSpcRN+ (89.2%), and exceeded those of nSc++ (48.6%) and nScRN++ (54.1%). Moreover, nSpcRN++‐ABE8e yielded the highest bi‐allelic ratio (Figure 6b). Similarly, at the *OsACC*‐I1879 target, the nSpcRN+‐ and nSpcRN++‐mediated ABEs exhibited superior mutation efficiency compared to nSc++ and nScRN++, and nSpcRN++‐based ABE8e demonstrated a higher bi‐allelic ratio than the others (Figure 6b). No indels were observed in the target regions of the mutant plants. Furthermore, our findings revealed that chimeric nSpcRN++‐ABE8e possessed an expansive base editing window. While nSc++‐ and nScRN++‐based ABE8es introduced A‐to‐G conversions at positions 6 and 8 within the main editing window (4–10) of the *OsACC*‐D2176 target, the A_11_ nucleotide situated outside the editing window was successfully edited by nSpcRN++‐ and nSpcRN++‐based ABEs (Figure 6c). Intriguingly, the base substitution induced by nSc++‐ and nScRN++‐based ABEs at A_6_ always occurred concurrently with that at A_8_. The desired singular A_6_‐to‐G_6_ conversion was observed exclusively with nSpcRN+‐ and nSpcRN++‐mediated base editing (Figure S9, Supporting Information). The nSpcRN++‐based ABE also exhibited the widest A_3_ to A_12_ editing window at the *OsACC*‐I1879 target (Figure 6c) and yielded the most diverse range of alleles (Figure S9, Supporting Information).

To evaluate the potential herbicide resistance conferred by amino acid changes at D2176 and I1879, we transferred the identified mutants to Kimura nutrient solution supplemented with 1.08 mg L^−1^ haloxyfop‐methyl. For the *OsACC*‐D2176 target, A_6_‐to‐G_6_ and A_8_‐to‐G_8_ would induce the D2176G and S2177G substitutions, respectively. Notably, only the mutants harboring a single D2176G mutation, generated by nSpcRN+‐ and nSpcRN++‐based ABE8es, survived the haloxyfop‐methyl treatment (Figure 6d). The lethality of the double D2176G/S2177G mutant suggests that the S2177G mutation may exert an antagonistic effect on the adjacent D2176G mutation. For the *OsACC*‐I1879 target, all viable mutants carried the I1879V substitution, and the mutation at N1878 appeared to have no detrimental effects on herbicide resistance (Figure 6e). The genotypes of the herbicide‐resistant mutants were further confirmed using Sanger sequencing (Figure S10, Supporting Information). Further comprehensive analyses are warranted to ascertain their herbicide resistance capabilities under field conditions. Taken together, SpcRN++ coupled with TadA8e represents a robust and efficient base editing tool with a wide editing window in rice, and holds promise for gain‐of‐function agricultural trait improvement.

## 3 Discussion

The necessity of PAM sequences constrains the application of CRISPR/Cas9 in genome editing to a certain extent. ScCas9 and its derivative Sc++, which recognize NNG PAM, have expanded the target range of genome editing tools.^[^ ^21^, ^22^ ^]^ Despite its high activity at NNG PAM targets in mammalian cells, the evolved Sc++ still exhibits strong PAM preferences and shows very low or no editing activity at NCG and NTG PAM targets in plants.^[^ ^23^, ^24^ ^]^ Given the high degree of sequence homology between ScCas9 and SpCas9, we hypothesized that modifications of SpCas9 could be used to optimize ScCas9. We found that the R221K and N394K mutations, which enhanced the nuclease activity of SpCas9, moderately increased the nuclease activity of Sc++ in rice protoplast cells (Figure 1b).

The REC domain of the Cas9 protein specifically recognizes and interacts with the guide RNA scaffold and the gRNA–DNA heteroduplex and plays a crucial role in triggering the conformational change necessary for activating the Cas9 nuclease domain to cleave the target DNA.^[^ ^43^, ^44^ ^]^ Previous studies have shown that replacing the guide RNA scaffold of ScCas9 with that of SpCas9 does not impact the editing efficiency of ScCas9.^[^ ^45^ ^]^ Considering that SpCas9 displays superior editing efficiency compared to Sc++ at NGG PAM targets in rice, we postulated that replacing the REC domain of Sc++ with that of SpCas9 could enhance its genome editing capabilities. Consequently, we engineered three chimeric Cas9 nucleases (Figure 1a). Among these, SpcRN++, comprising the R221K/N394K/T1227K mutations, a positively charged loop, and the REC domain of SpCas9, outperformed other variants at diverse genomic loci with NNG PAMs in both transient protoplast assays and transgenic plants. These findings suggest that these modifications have a synergistic effect on the nuclease activity of ScCas9. Increasing the affinity of Cas9 nuclease could compensate for poor targeting sites and ultimately enable editing at suboptimal PAM sites. The results of this study support this hypothesis. While chimeric SpcRN++ exhibited a slight increase in editing efficiency at the more preferred NAG and NGG PAM targets, it showed significantly enhanced editing efficiencies at the less preferred NTG PAM targets (Figure 1c and Figure 2a). Therefore, the SpcRN++ developed in this study has promising applications in plants because of its wide target range and increased editing activity.

The improved nuclease activity of Cas9 may result in an increased incidence of protospacer‐dependent off‐target effects. The mismatch tolerance assay indicated that SpcRN++ exhibited higher tolerance than Sc++ to certain single mismatches outside the seed region (Figure 3). Notably, no off‐target mutations introduced by the four ScCas9 based variants were detected at any of the predicted off‐target sites in the transgenic plants. These findings indicate that the enhanced nuclease activity of SpcRN++ may contribute to off‐target effects, but can be eliminated by carefully designing specific protospacers.

The editing window of the base editors was significantly constrained by the PAM sequences. Therefore, we evaluated the compatibility of SpcRN++ with cytosine and adenine base editing. Our findings indicated that the enhanced genome editing efficiency of SpcRN++ also contributed to boosting C‐to‐T editing rates across all tested targets in rice protoplasts. Furthermore, nSpcRN++‐ABE8e demonstrated increased A‐to‐G conversion efficiencies at half of the tested sites while exhibiting comparable efficiencies at other targets in protoplasts. Notably, this enhancement was predominantly observed for targets with a lower editing efficiency (Figure 4c). This was supported by the fact that TadA8e already possesses an intrinsic hyperactive adenosine deamination capacity, and that enhanced activity in SpCas9 only increases ABE efficiencies at suboptimal targets in mammalian cells.^[^ ^27^ ^]^ Moreover, the chimeric nSpcRN+‐ and nSpcRN++‐based ABE8es exhibited significantly higher efficiencies than nSc++ and nScRN++ in T_0_ transgenic plants at two tested targets, suggesting that the former may be more capable of targeting adenine bases outside the typical editing window, result in a wider editing window in transgenic plants. Further studies are required to fully understand the wide editing window induced by nSpcRN++‐ABE8e in stable transgenic rice plants.

Acetyl‐coenzyme A carboxylase (ACCase) is a key target enzyme for a group of commercial herbicides. Almost all known ACCase‐inhibiting herbicide‐resistant mutations reside within its carboxyl transferase (CT) domain. Various genome editing tools, including CBEs, ABEs, dual BE, and prime editing, have been extensively used to alter specific amino acids or artificially evolve the *OsACC* gene to produce herbicide‐resistant rice.^[^ ^46^, ^47^, ^48^, ^49^ ^]^ To date, 25 amino acid substitutions at 13 sites in *OsACC* that confer herbicide resistance have been identified.^[^ ^50^ ^]^ However, these genome editing tools are primarily based on SpCas9, which limits their target range. For example, because of the PAM requirement and the editing window of SpCas9‐derived base editors, no proper base editing vectors can be designed to target D2176 amino acid. Although the D2176G mutation was discovered through the prime editing‐library mediated saturation mutagenesis (PLSM) method, only one heterozygous mutant was obtained, likely due to the low efficiency of prime editing. The heterozygous D2176G mutant died after being transplanted into soil, preventing the evaluation of herbicide‐resistance at the seedling stage.^[^ ^46^ ^]^ In this study, we successfully generated several herbicide‐resistant rice lines harboring either homozygous or heterozygous D2176G substitutions using the nSpcRN++‐ABE8e system. Considering their enhanced activity and broad target range, SpcRN++‐based genome editing tools may facilitate the screening of additional herbicide‐resistant sites.

Protein engineering is a powerful tool for redesigning natural proteins and for creating new artificial proteins. A variety of engineering methods encompassing rational and semi‐rational design, as well as directed evolution, have been employed to enhance the capabilities of existing genome editing tools. This has led to improvements in several key areas, including increased editing activity, reduced off‐target effects, and flexible PAM requirements. The Sc++ variant, which exhibited enhanced editing activity relative to ScCas9, was rationally designed by incorporating crucial amino acids computationally identified from *Streptococcus* orthologs.^[^ ^22^ ^]^ Directed evolution is an effective method for creating new Cas nuclease variants without prior knowledge of target molecules. Previously, R221K/N394K mutations were identified by deep mutational scanning as being capable of elevating the nuclease activity of SpCas9.^[^ ^31^ ^]^ We further demonstrated that the combination of homologous R221K/N394K mutations in ScRN++ resulted in a slight enhancement in the editing efficiency of Sc++ (Figure 1b). Structure‐guided modular chimeragenesis offers additional possibilities for generating non‐natural synthetic proteins with altered characteristics. For example, a chimeric SpRYc variant, created by grafting the PAM‐interacting domain of SpRY to the N‐terminus of Sc++, exhibited robust PAM flexibility.^[^ ^33^ ^]^ Notably, the current investigations did not include the REC domain. In this study, a novel SpcRN++ variant was engineered by combining the advantages of rationally designed Sc++, evolutionary R221K/N394K mutations, and the chimeric REC domain of SpCas9. Our study illustrates the potential of integrating diverse protein engineering approaches and paves the way for future advances in Cas protein design.

## 4 Experimental Section

### Plasmid Construction

The ScCas9 sgRNA scaffold was chemically synthesized by GenScript (Nanjing, China) and subsequently fused to the *OsU3* promoter using overlap PCR. The generated OsU3‐Sc‐sgRNA cassette was used to replace the OsU3‐Sp‐sgRNA cassette in pHUE411^[^ ^51^ ^]^ via *Hin*dIII digestion, resulting in the pHUE‐Sc‐sg vector. Additionally, rice codon‐optimized Sc++ sequences were synthesized and subcloned to replace the *SpCas9* gene in the pHUE‐Sc‐sg vector using *Xma*JI/*Sac*I double digestion to generate the binary vector pHUE‐Sc++. Subsequently, the R221K and N394K mutations were introduced into the *Sc++* gene by segmented PCR amplification and simultaneously inserted into an *Xma*JI/*Sac*I pre‐digested pHUE‐Sc‐sg vector using Gibson assembly to produce the binary vector pHUE‐ScRN++. An analogous approach was employed to create chimeric *Spc+*, *SpcRN+*, and *SpcRN++* genes by segmented PCR amplification, which were inserted into the *Xma*JI/*Sac*I pre‐digested pHUE‐Sc‐sg vector using the Gibson assembly. The detailed amino acid sequences of Sc++, ScRN++, Spc+, SpcRN+, and SpcRN++ are presented in the Sequences (Supporting Information). For ABE8e constructs, a D10A mutation was introduced into Sc++, ScRN++, Spc+, SpcRN+, and SpcRN++ by site‐specific mutagenesis. A3A/Y130Y and multiple copies of UGI amplified from SpG‐eA3A,^[^ ^52^ ^]^ combined with nSc++/nScRN++/nSpcRN+/nSpcRN++ were inserted into the pHUE‐Sc‐sg vector using the Gibson assembly. TadA‐8e amplified from SpG‐ABE8e,^[^ ^52^ ^]^ nSc++/nScRN++/ nSpcRN+/nSpcRN++, and 3×NLS were inserted in an orderly manner into the pHUE‐Sc‐sg vector using the Gibson assembly. To construct sgRNAs for single knockout and base editing, pairs of oligonucleotides for each sgRNA were synthesized, annealed, and cloned into the corresponding *Bsa*I‐digested plasmids. The pCBCMT1T2 vector was used as the PCR template to construct the two sgRNAs.^[^ ^51^ ^]^

### Protoplast Transfection

Rice protoplast isolation and PEG‐mediated transfection were performed as previously described.^[^ ^53^ ^]^ In brief, the Japonica rice variety Nipponbare was cultivated in half‐strength MS medium at 28 °C with a photoperiod of 16 h light: 8 h dark for 2 weeks to facilitate protoplast preparation. Twenty micrograms of each plasmid were delivered into protoplast cells. Transfected protoplasts were incubated at 23 °C in the dark for 48 h. Subsequently, the protoplasts were collected, and genomic DNA was extracted using the CTAB method^[^ ^54^ ^]^ for amplicon deep sequencing.

### Amplicon Deep Sequencing for Protoplast Assay

Two rounds of PCR were performed to prepare a library for sequencing. In the first round, target regions were amplified using site‐specific primers (Table S4, Supporting Information). In the second round, both forward and reverse barcodes were added to the 5′‐termini of the primer sets, limiting the PCR product size to 300 bp (Table S4, Supporting Information). Typically, up to 80 distinct PCR products can be pooled together, each containing ≈300 ng, and then commercially sequenced using the NovaSeq PE150 platform (Novogene). Three independent biological replicates were employed for each target site. Mutation efficiencies were analyzed using the CRISPRMatch software.^[^ ^55^ ^]^

### Agrobacterium‐Mediated Transformation

All binary constructs were transformed into *Agrobacterium tumefaciens* strain EHA105 using the freeze/thaw method and selected on LB medium supplemented with kanamycin (50 mg L^−1^) and rifampicin (25 mg L^−1^). Positive clones were confirmed by Sanger sequencing of the target regions. Rice transformation was performed as described previously by Hiei et al.,^[^ ^56^ ^]^ Mature seeds of the Japonica rice variety Nipponbare were used for callus induction. Embryogenic calli were then subjected to infection by *Agrobacterium* and selected using 50 mg L^−1^ hygromycin for 4 weeks to obtain resistant calli. The surviving calli were transferred to regeneration medium containing 50 mg L^−1^ hygromycin to regenerate the transgenic plants.

### Genotyping of T_0_ Transgenic Plants

Three to four leaves from each seedling were collected as a single sample. Genomic DNA from each seedling was extracted using the CTAB method and used as the PCR template. Target sequences were amplified using specific primers (Table S4, Supporting Information) and detected using high‐throughput tracking of mutation (Hi‐TOM) assays. Mutation reads lower than 3% for knockout and 10% for base editing were filtered during data analysis.

### Off‐Target Analysis

The potential off‐target sites were predicted using the CRISPR‐GE online tool. Nine candidate off‐target sites with one to three base mismatches against three targets (*OsIPA1*, *OsWSL1*, and *OsSPP2*) for the Sc++, ScRN+, SpcRN+, and SpcRN++ tools were selected for this study. PCR products encompassing putative off‐target regions were amplified and subsequently analyzed using commercial Hi‐TOM sequencing.

### Screening for Herbicide Tolerance

To analyze herbicide resistance induced by the A‐to‐G conversions at the *OsACC*‐D2176 and I1879 targets, the mutants identified by Hi‐TOM sequencing were transferred to 1× Kimura nutrient solution containing 1.08 mg L^−1^ haloxyfop‐methyl, and incubated at 28 °C with a photoperiod of 16 h light: 8 h dark. Photographs were taken after 10 days of treatment.

### Statistical Analysis

All data presented in this study for the protoplast assay were derived from three biological replicates. Statistical analyses were performed and graphical representations were created using GraphPad Prism software (version 9). Data are expressed as the mean ± standard error of the mean (s.e.m.), with individual data points denoted by dots. Statistical differences between the control and the treatments were tested using two‐tailed Student's *t*‐tests. The significance levels were defined as follows: ^*^ *p* < 0.05, ^**^ *p* < 0.01, ^***^ *p* < 0.001, and ^****^ *p* < 0.0001.

## Conflict of Interest

The authors have submitted patent applications based on the results of this study.

## Author Contributions

Z.L. and Y.W. contributed equally to this work. Z.L., Y.G., and Y.W. designed the projects; Z.L., Y.W., and S.D. performed most of the experiments; Y.G. analyzed the amplicon deep sequencing data; S.W. and K.Z. performed rice transformation work; Z.L. and Y.W. wrote the manuscript and Z.L. supervised the project.

## Supporting information



Supporting Information

## Data Availability

The data that support the findings of this study are available from the corresponding author upon reasonable request.
