# Supplementary material for: Enhanced Genome Editing Activity with Novel Chimeric ScCas9 Variants in Rice
Source: Adv Sci (Weinh). 2025 Jan 4;12(8):2411549. doi: 10.1002/advs.202411549 (PMC11848528; doi:10.1002/advs.202411549)
Supplement: Supplementary file 1 — Supporting Information [file ADVS-12-2411549-s001.docx]

Supporting Information

**Enhanced Genome Editing Activity with Novel Chimeric ScCas9 Variants in Rice**

*Zhen Liang^*^, Yuqing Wu, Shuke Deng, Sha Wei, Kai Zhang and Yingjie Guo^*^*

This Supporting Information file includes:

Figure S1 to Figure S10

Table S1 to Table S4


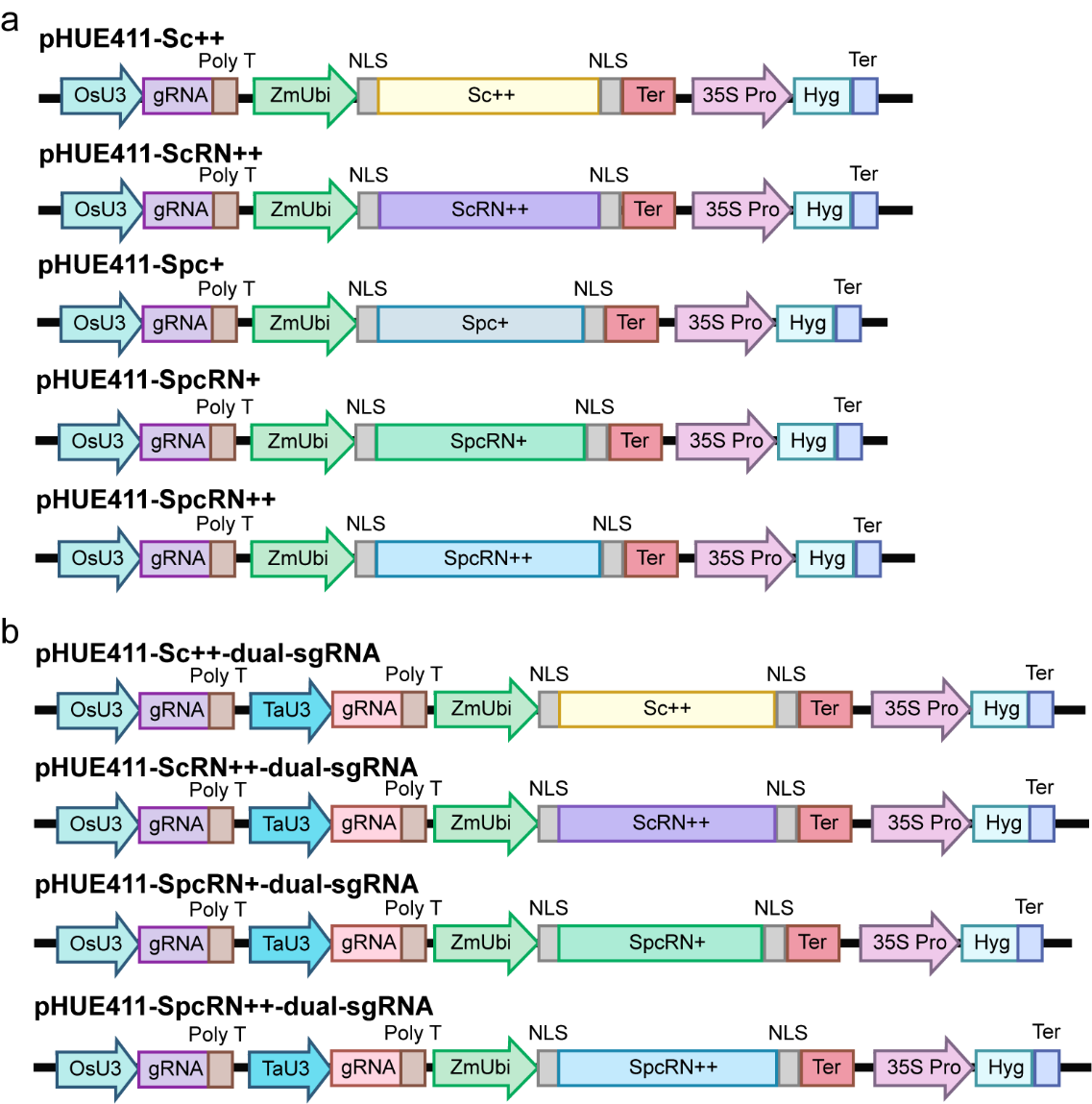


**Figure S1. Schematic illustration of knockout constructs with Sc++, ScRN++, Spc+, SpcRN+ and SpcRN++ variants. (a)** Single knockout with *OsU3* driven sgRNA cassette for rice protoplast assay. **(b)** Double knockout with *OsU3* and *TaU3* driven sgRNA cassettes for stable transgenic rice plants.


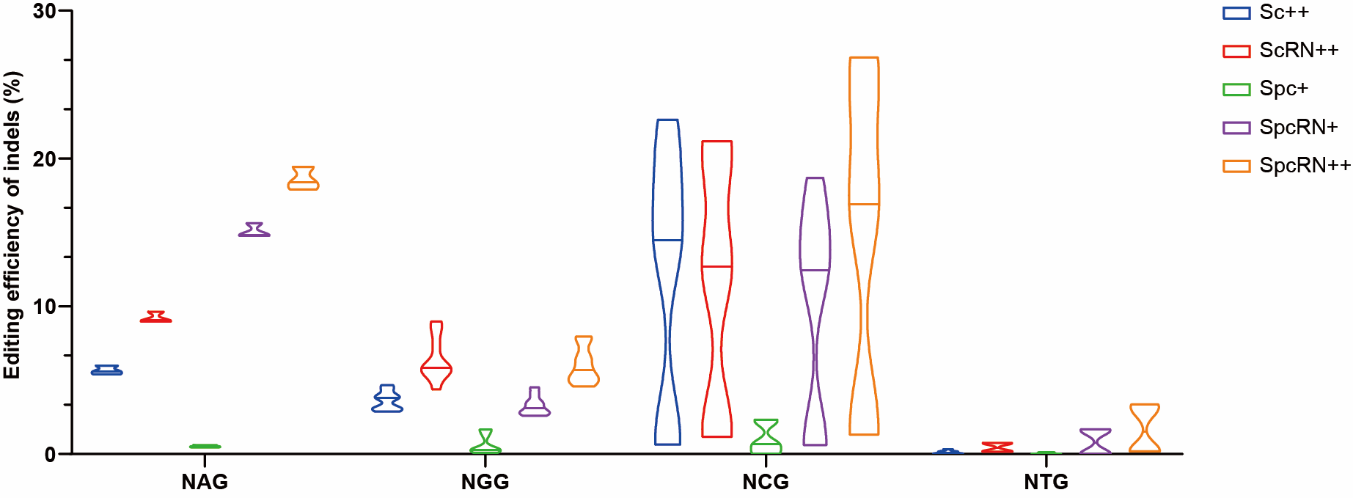


**Figure S2. Overall editing efficiencies induced by Sc++ based variants in rice protoplasts at NGG, NCG, NTG, and NAG PAM targets.**

**
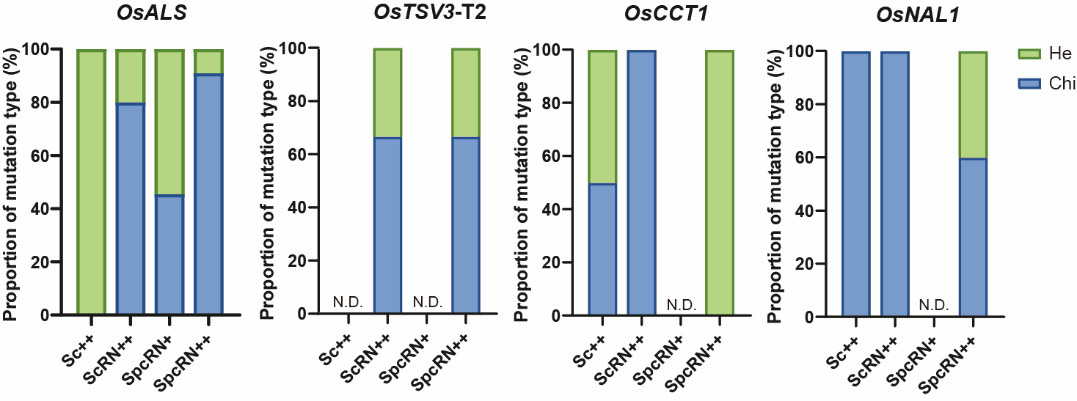
**

**Figure S3. Mutation patterns and ratios induced by Sc++, ScRN++, SpcRN+ and SpcRN++ at four target sites in T_0_ transgenic plants.**

**
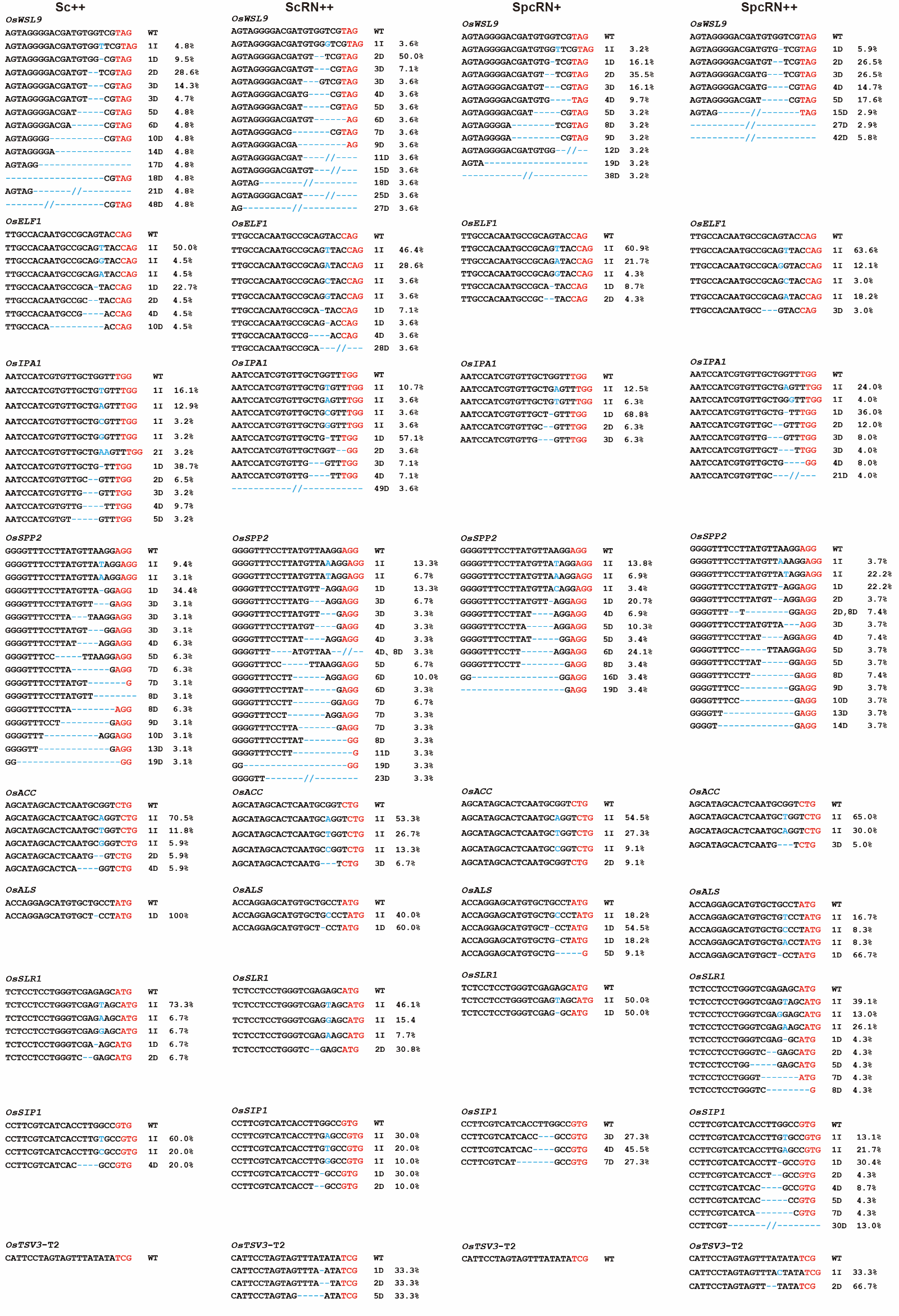
**

**
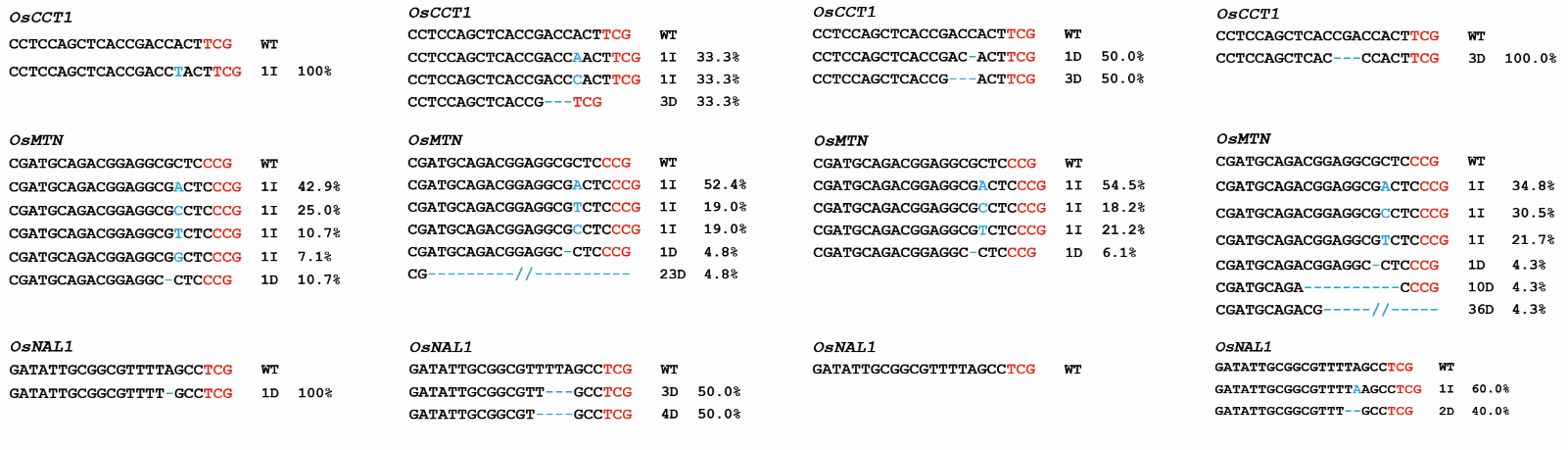
**

**Figure S4. Mutation types at 12 target sites induced by Sc++ and its variants in T_0_ transgenic plants.**

**
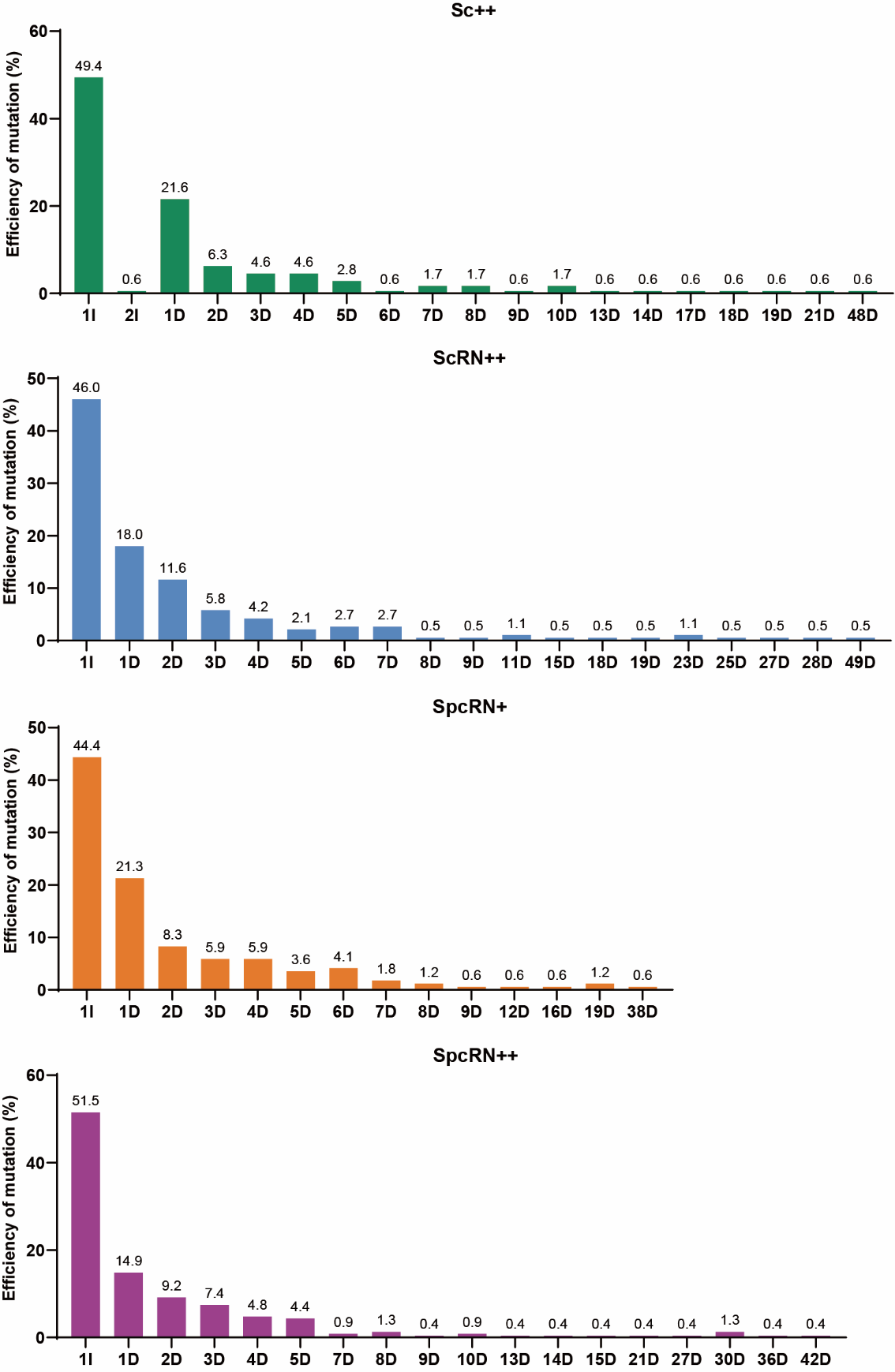
**

**Figure S5. Summary of the mutation types induced by Sc++ and its variants in T_0_ transgenic plants.**

**
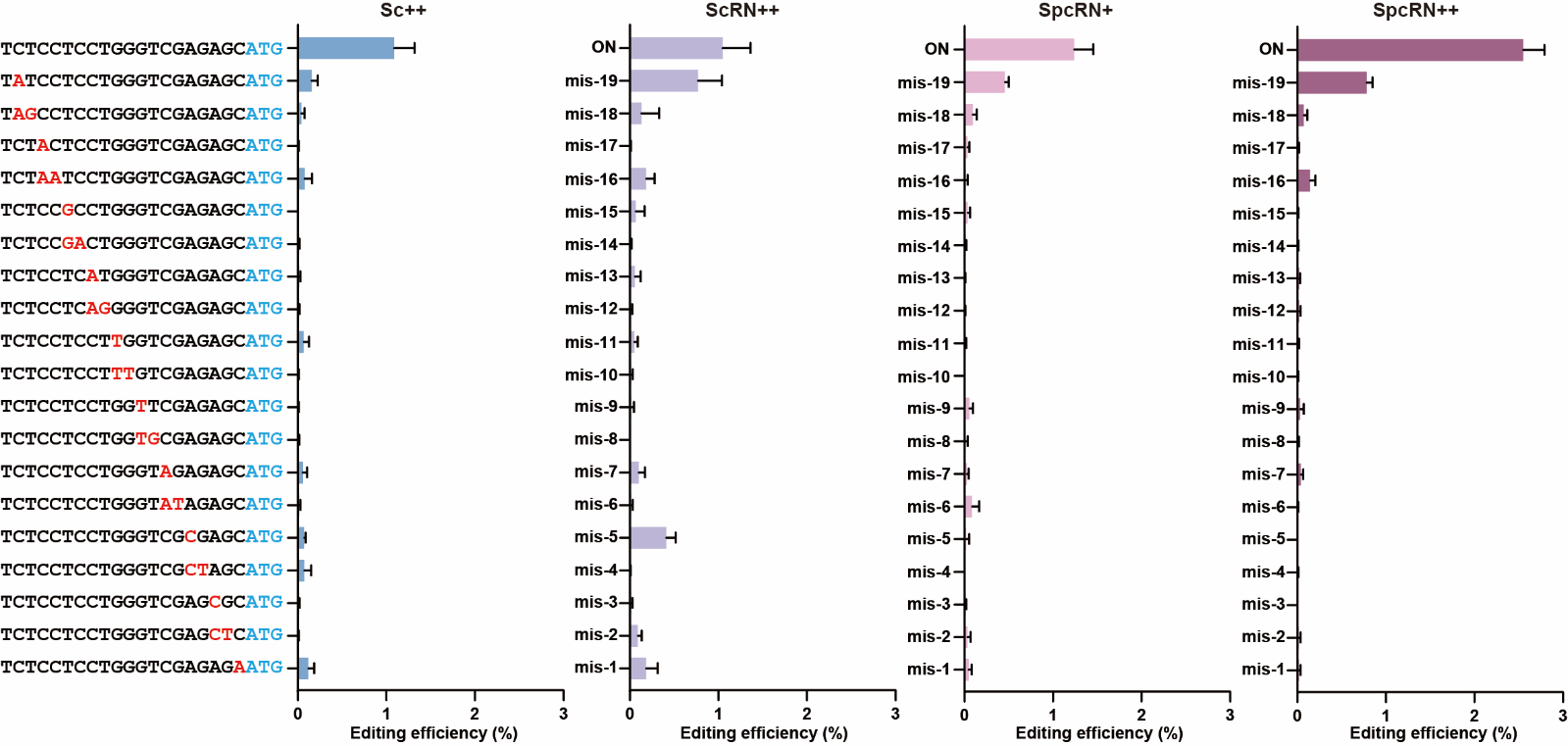
**

**Figure S6. Comparisons of the sequence specificities of Sc++, ScRN++, SpcRN+, and SpcRN++ at the *OsSLR1* target.** Off-target effects of Sc++ and its variants ScRN++, SpcRN+, and SpcRN++ with guide sequences containing singular or double mismatches at successive positions against the *OsSLR1* target. Each sgRNA was tested in combination with the four ScCas9 variants, and the percentage of indels was used to measure editing activity. Editing efficiencies (mean ± s.e.m.) are based on three independent experiments (*n* = 3). The WT guide sequence is highlighted in bold with the PAM highlighted in blue. The mismatch sites are highlighted in red.


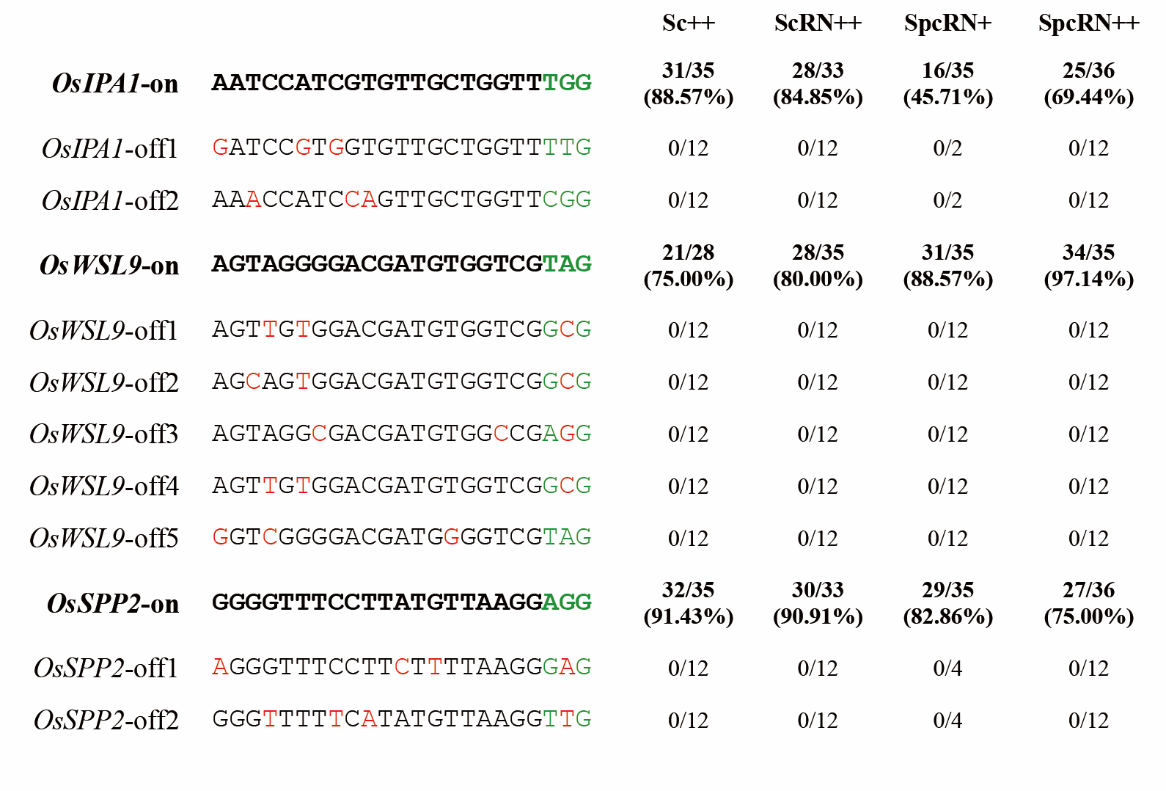


**Figure S7. Detection of the off-target effects in T_0_ transgenic plants.**


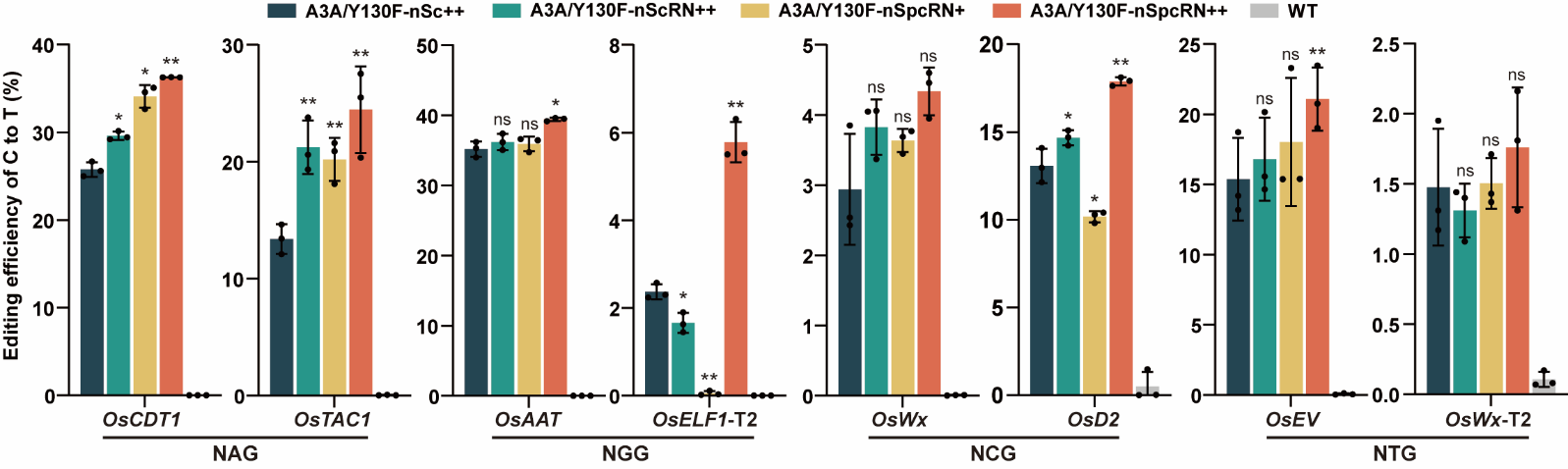


**Figure S8. Comparison of the cytosine base editors based on the four ScCas9 variants in rice protoplasts.** These efficiencies were defined as the highest C-to-T conversions of the multiple Cs within the editing window. Editing efficiencies (mean ± s.e.m.) are based on three independent experiments (n = 3). *P-values* were determined using two-tailed Student’s t-test: ns, *p* > 0.05, **p* < 0.05, ***p* < 0.01.

**
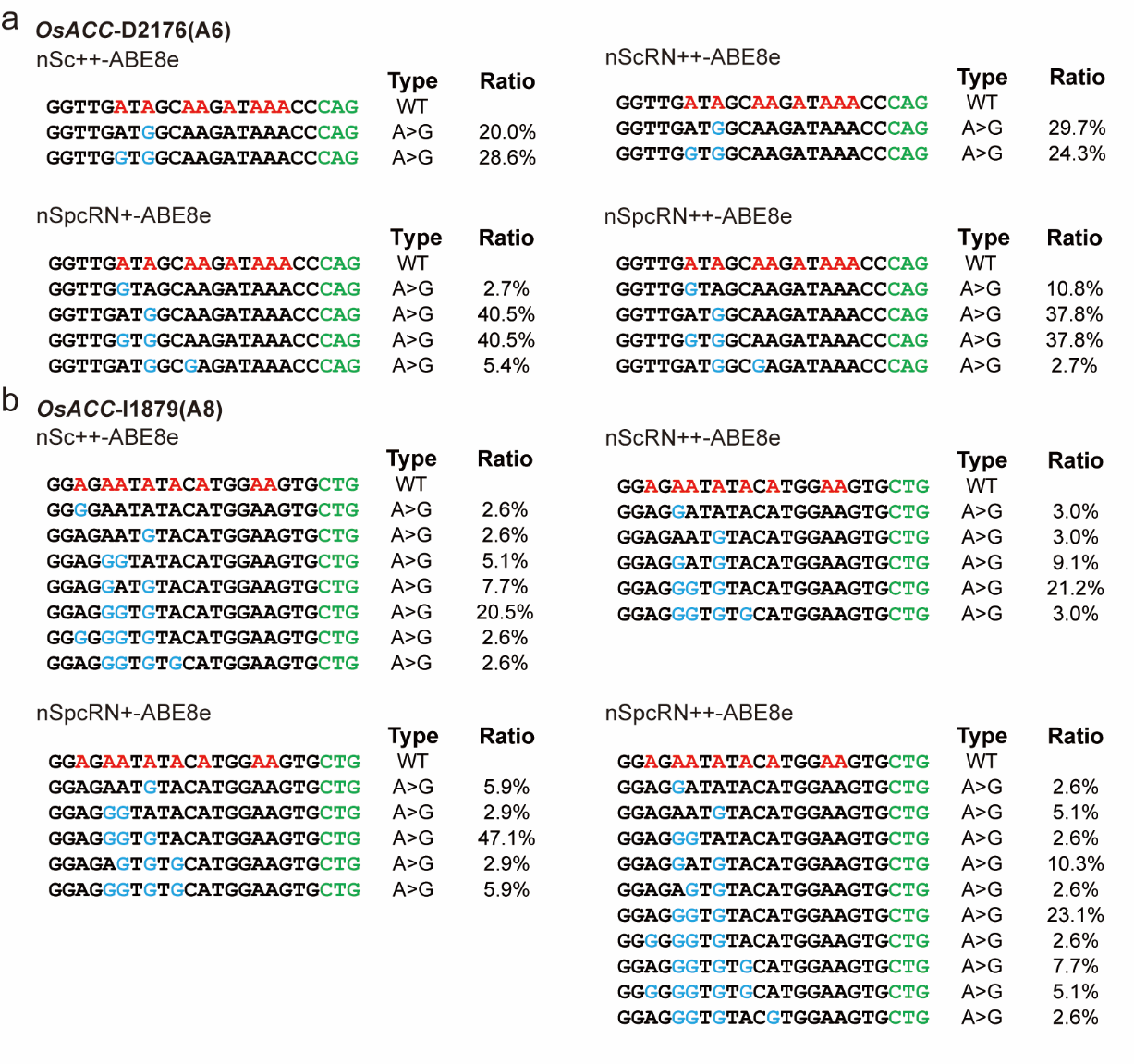
**

**Figure S9. Genotyping of the T_0_ transgenic plants induced by Sc++ and its variants based ABE at the *OsACC*-D2176 and *OsACC*-I1879 targets.**

**
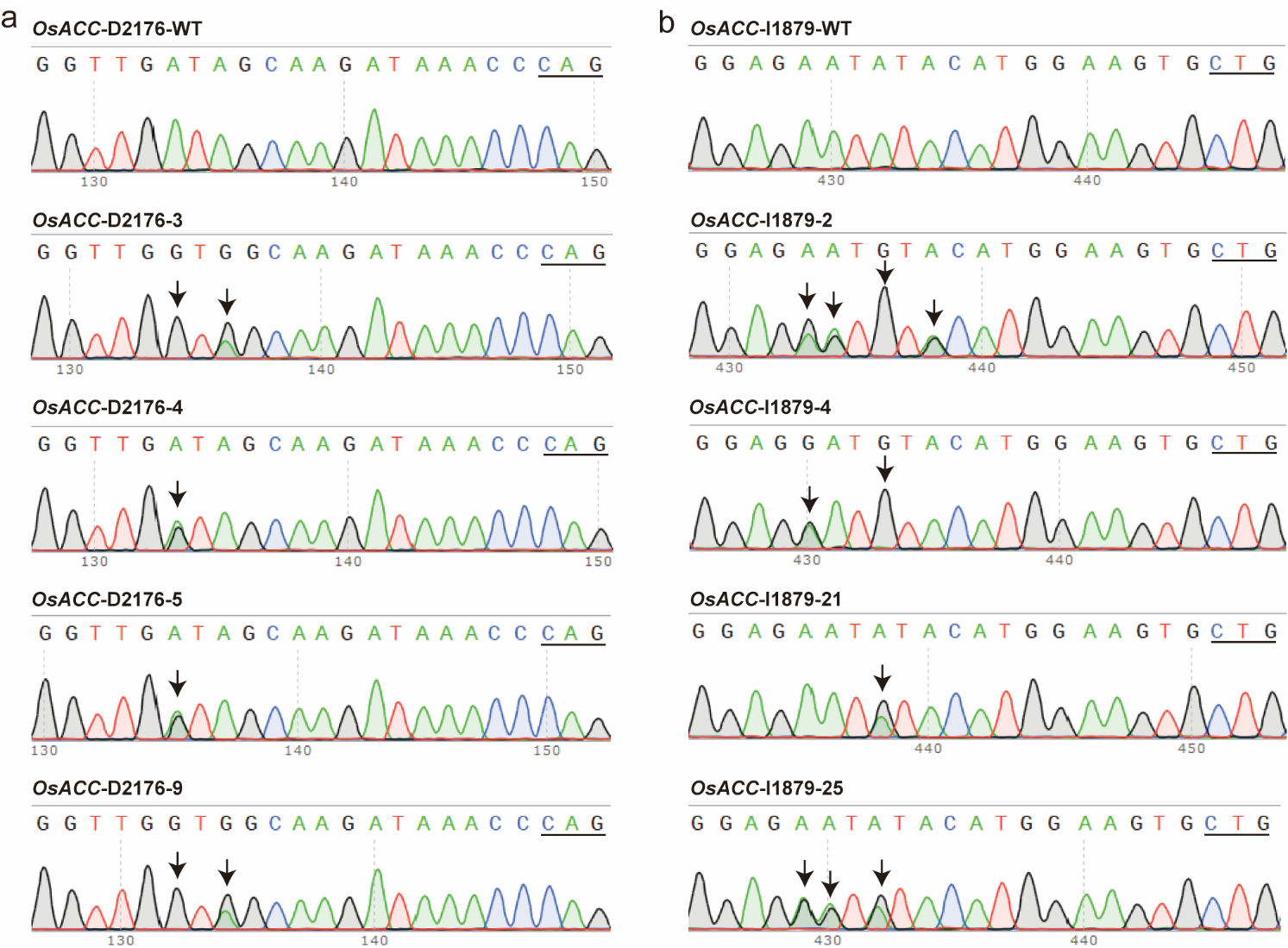
**

**Figure S10. Sanger-****sequencing chromatograms of herbicide resistant lines harboring the desired mutations.** The targeted A-to-G mutations are indicated by black arrows and the PAM sequences are underlined.

**Table S1.** sgRNA target site.

| Target | Gene ID | PAM | Spacer | Application |
| --- | --- | --- | --- | --- |
| *OsHOSS66* | LOC_Os03g03164 | NAG | CGATCCCTCATGGAGCGGGTCAG | knockout |
| *OsGAPDH* | LOC_Os08g34210 |  | TCCCGGGCAATGAACGGAACAAG |  |
| *OsROC4* | LOC_Os04g48070 |  | CCCATCTGAGCCATCTTGACTAG |  |
| *OsWSL9* | LOC_Os03g07370 |  | AGTAGGGGACGATGTGGTCGTAG |  |
| *OsELF1* | LOC_Os03g13010 |  | TTGCCACAATGCCGCAGTACCAG |  |
| *OsGAPDH*-T2 | LOC_Os08g34210 | NGG | GATTGCAGAGTGCCTCGTCAAGG |  |
| *OsADH1* | LOC_Os11g10480 |  | TTGTCTGATTAGGCCGCAGAAGG |  |
| *OsHDS* | LOC_Os02g39160.1 |  | GGTGGGCTCATCAAGAGGCAGGG |  |
| *OsIPA1* | LOC_Os08g39890 |  | AATCCATCGTGTTGCTGGTTTGG |  |
| *OsSPP2* | LOC_Os01g27880 |  | GGGGTTTCCTTATGTTAAGGAGG |  |
| *OsBADH2* | LOC_Os08g32870 |  | GACTAGAGACGCTTGATTGTGGG |  |
| *OsEPSPS* | LOC_Os06g04280 | NCG | GCCTTACTGAGCTGAATTATTCG |  |
| *OsHPPD* | LOC_Os02g07160 |  | AGCGGCGGAGCCAGATACAGACG |  |
| *OsCPK6* | LOC_Os02g58520 |  | GGGCAACTACTACTCGTGCGGCG |  |
| *OsTSV3* | LOC_Os03g58540.1 |  | ATCTTAAGGGTGGTTGCAGACG |  |
| *OsTSV3*-T2 | LOC_Os03g58540.1 |  | CATTCCTAGTAGTTTATATATCG |  |
| *OsTGW2* | LOC_Os02g52550 |  | GAGGGCTCAGTTCGACCTGGACG |  |
| *OsCCT1* | LOC_Os01g61900 |  | CCTCCAGCTCACCGACCACTTCG |  |
| *OsNAL1* | LOC_Os04g52479 |  | GATATTGCGGCGTTTTAGCCTCG |  |
| *OsSULTR* | LOC_Os01g52130 | NTG | ACTTGGGACAGGTCGGTAACCTG |  |
| *OsLRR* | LOC_Os11g07225 |  | CAGCGAGGTACCAATGCCGGATG |  |
| *OsFBK1* | LOC_Os01g47050 |  | TATGACTCACAGAGTAGTTCATG |  |
| *OsSLR1* | LOC_Os03g49990 |  | TCTCCTCCTGGGTCGAGAGCATG |  |
| *OsSIP1* | LOC_Os01g08660.1 |  | CCTTCGTCATCACCTTGGCCGTG |  |
| *OsACC* | LOC_Os05g22940 |  | AGCATAGCACTCAATGCGGTCTG |  |
| *OsALS* | LOC_Os02g30630 |  | ACCAGGAGCATGTGCTGCCTATG |  |
| *OsCDT1* | LOC_Os03g45370 | NAG | GCATAGCAACACCCCCCACCTAG | Cytosine base editor  (A3A-BE) |
| *OsTAC1* | LOC_Os09g35980 |  | ACAAATCCCGCAAAAGGTGAAAG |  |
| *OsAAT* | LOC_Os01g55540 | NGG | CAAGGATCCCAGCCCCGTGAAGG |  |
| *OsELF1*-T2 | LOC_Os03g13010 |  | TGCCACAATGCCGCAGTACCAGG |  |
| *OsWx* | LOC_Os06g04200 | NCG | GAGCACATTCTCCCAGTTCTTCG |  |
| *OsD2* | LOC_Os01g10040 |  | ACTCCGTCCCGGTGCTCATCACG |  |
| *OsEV* | LOC_Os02g11010 | NTG | GCACACACACACTAGTACCTCTG |  |
| *OsWx*-T2 | LOC_Os06g04200 |  | CCCCAGCCGCTTCGAGCCCTGTG |  |
| *OsGS1* | LOC_Os02g50240 | NAG | TGCTCACACCAACTACAGGTGAG | Adenine Base editing (ABE8e) |
| *OsWSL9* | LOC_Os03g07370 |  | AGTAGGGGACGATGTGGTCGTAG |  |
| *OsACC*-D2176G | LOC_Os05g22940 |  | GGTTGATAGCAAGATAAACCCAG |  |
| *OsIPA1*-T2 | LOC_Os08g39890 | NGG | AGAGAGAGCACAGCTCGAGTCGG |  |
| *OsALS*-T2 | LOC_Os02g30630 |  | CCCAAGTGGGGGCGCATTCAAGG |  |
| OsMTN | LOC_Os06g02220 | NCG | CGATGCAGACGGAGGCGCTCCCG |  |
| *OsTubA2* | LOC_Os11g14220 |  | CCACTTCATGCTTTCCTCCTACG |  |
| *OsACC*-T2 | LOC_Os05g22940 | NTG | AGCATAGCACTCAATGCGGTCTG |  |
| *OsACC*-T3(I1879V) | LOC_Os05g22940 |  | GGAGAATATACATGGAAGTGCTG |  |

The PAMs in each target sequence are highlighted in red.

**Table S2.** Mismatched sgRNA target site.

| Target | Spacer | Mismatch site | Type |
| --- | --- | --- | --- |
| *OsWSL9* | AGTAGGGGACGATGTGGTCGTAG | WT | C>A or A>C or G>T or T>G |
| *OsWSL9*-mis-1 | AGTAGGGGACGATGTGGTCTTAG | 1 |  |
| *OsWSL9*-mis-2 | AGTAGGGGACGATGTGGGAGTAG | 2+3 |  |
| *OsWSL9*-mis-3 | AGTAGGGGACGATGTGGGCGTAG | 3 |  |
| *OsWSL9*-mis-4 | AGTAGGGGACGATGTTTTCGTAG | 4+5 |  |
| *OsWSL9*-mis-5 | AGTAGGGGACGATGTTGTCGTAG | 5 |  |
| *OsWSL9*-mis-6 | AGTAGGGGACGATTGGGTCGTAG | 6+7 |  |
| *OsWSL9*-mis-7 | AGTAGGGGACGATTTGGTCGTAG | 7 |  |
| *OsWSL9*-mis-8 | AGTAGGGGACGCGGTGGTCGTAG | 8+9 |  |
| *OsWSL9*-mis-9 | AGTAGGGGACGCTGTGGTCGTAG | 9 |  |
| *OsWSL9*-mis-10 | AGTAGGGGAATATGTGGTCGTAG | 10+11 |  |
| *OsWSL9*-mis-11 | AGTAGGGGAAGATGTGGTCGTAG | 11 |  |
| *OsWSL9*-mis-12 | AGTAGGGTCCGATGTGGTCGTAG | 12+13 |  |
| *OsWSL9*-mis-13 | AGTAGGGTACGATGTGGTCGTAG | 13 |  |
| *OsWSL9*-mis-14 | AGTAGTTGACGATGTGGTCGTAG | 14+15 |  |
| *OsWSL9*-mis-15 | AGTAGTGGACGATGTGGTCGTAG | 15 |  |
| *OsWSL9*-mis-16 | AGTCTGGGACGATGTGGTCGTAG | 16+17 |  |
| *OsWSL9*-mis-17 | AGTCGGGGACGATGTGGTCGTAG | 17 |  |
| *OsWSL9*-mis-18 | ATGAGGGGACGATGTGGTCGTAG | 18+19 |  |
| *OsWSL9*-mis-19 | ATTAGGGGACGATGTGGTCGTAG | 19 |  |
| *OsSLR1* | TCTCCTCCTGGGTCGAGAGCATG | WT | C>A or A>C or G>T or T>G |
| *OsSLR1*-mis-1 | TCTCCTCCTGGGTCGAGAGAATG | 1 |  |
| *OsSLR1*-mis-2 | TCTCCTCCTGGGTCGAGCTCATG | 2+3 |  |
| *OsSLR1*-mis-3 | TCTCCTCCTGGGTCGAGCGCATG | 3 |  |
| *OsSLR1*-mis-4 | TCTCCTCCTGGGTCGCTAGCATG | 4+5 |  |
| *OsSLR1*-mis-5 | TCTCCTCCTGGGTCGCGAGCATG | 5 |  |
| *OsSLR1*-mis-6 | TCTCCTCCTGGGTATAGAGCATG | 6+7 |  |
| *OsSLR1*-mis-7 | TCTCCTCCTGGGTAGAGAGCATG | 7 |  |
| *OsSLR1*-mis-8 | TCTCCTCCTGGTGCGAGAGCATG | 8+9 |  |
| *OsSLR1*-mis-9 | TCTCCTCCTGGTTCGAGAGCATG | 9 |  |
| *OsSLR1*-mis-10 | TCTCCTCCTTTGTCGAGAGCATG | 10+11 |  |
| *OsSLR1*-mis-11 | TCTCCTCCTTGGTCGAGAGCATG | 11 |  |
| *OsSLR1*-mis-12 | TCTCCTCAGGGGTCGAGAGCATG | 12+13 |  |
| *OsSLR1*-mis-13 | TCTCCTCATGGGTCGAGAGCATG | 13 |  |
| *OsSLR1*-mis-14 | TCTCCGACTGGGTCGAGAGCATG | 14+15 |  |
| *OsSLR1*-mis-15 | TCTCCGCCTGGGTCGAGAGCATG | 15 |  |
| *OsSLR1*-mis-16 | TCTAATCCTGGGTCGAGAGCATG | 16+17 |  |
| *OsSLR1*-mis-17 | TCTACTCCTGGGTCGAGAGCATG | 17 |  |
| *OsSLR1*-mis-18 | TAGCCTCCTGGGTCGAGAGCATG | 18+19 |  |
| *OsSLR1*-mis-19 | TATCCTCCTGGGTCGAGAGCATG | 19 |  |

The PAMs in each target sequence are shown in red. The mismatches are shown in blue.

**Table S3**. The sgRNA spacer sequence-like off-target sites identified by CRISPR-GE used in T_0_ rice plants.

| Target | On- or Off-target | Spacer with PAM sequence | Locus on genome | Number of mismatch |
| --- | --- | --- | --- | --- |
| *OsIPA1* | *OsIPA1*-on | AATCCATCGTGTTGCTGGTTTGG | Chr8:25274448-25278696 | 0 |
|  | *OsIPA1*-off1 | GATCCGTGGTGTTGCTGGTTTTG | Chr2:35056929-35062529 | 3 |
|  | *OsIPA1*-off2 | AAACCATCCAGTTGCTGGTTCGG | Chr2:35540296-35543492 | 3 |
| *OsWSL9* | *OsWSL9*-on | AGTAGGGGACGATGTGGTCGTAG | Chr3:3744426-3746086 | 0 |
|  | *OsWSL9*-off1 | AGTTGTGGACGATGTGGTCGGCG | Chr11:20223784-20223806 | 2 |
|  | *OsWSL9*-off2 | AGCAGTGGACGATGTGGTCGGCG | Chr2:4922208-4922231 | 2 |
|  | *OsWSL9*-off3 | AGTAGGCGACGATGTGGCCGAGG | Chr9:18469701-18474199 | 2 |
|  | *OsWSL9*-off4 | AGTTGTGGACGATGTGGTCGGCG | Chr10:4823711-4823733 | 2 |
|  | *OsWSL9*-off5 | GGTCGGGGACGATGGGGTCGTAG | Chr7:25760179-25760202 | 3 |
| *OsSPP2* | *OsSPP2*-on | GGGGTTTCCTTATGTTAAGGAGG | Chr1:15568775-15573377 | 0 |
|  | *OsSPP2*-off1 | AGGGTTTCCTTCTTTTAAGGGAG | Chr9:15208404-15215467 | 3 |
|  | *OsSPP2*-off2 | GGGTTTTTCATATGTTAAGGTTG | Chr1:5559547-5568844 | 3 |

The PAMs are in red and the mismatched bases are in green.

**Table S4**. PCR primers used in this study.

| Prime name | Prime sequence | Application |
| --- | --- | --- |
| M13-F | TGTAAAACGACGGCCAGT | Constructed the pHUE411-Sc-sg vector |
| OsU3F-R | TAGGTACCTAGCTCTAAAACTGAGACCTTGTGTTGGTCTC |  |
| Sc-sg-F | GTTTTAGAGCTAGGTACCTAGC |  |
| Sc-sg-R | AGAAAACTCAATGCAAAACGAAAAAAAAAAGCACCGACTCGGTGCCACTT |  |
| gRNA-HindIII-F | TTTTTTTTTTCGTTTTGCATTGAGTTTTCT |  |
| HindIII-R | CTGCACTGCAGGCATGCAAG |  |
| 411-Sc-F1 | TTTGGTGTTACTTCTGCAGCCCTAGGATGGCTCCGAAGAAGAAGAGGAAGGTT | Amplified Sc++ variants |
| 411-Sc-R1 | CGAACGAAAGCTCTGAGCTCTCAGTGGTGGTGGTGGTGGTGCTTCTTCTTCTTCGCCTG |  |
| ScR221K-R | CTTCTCGAGCTTCTTGGACT |  |
| ScR221K-F | AGTCCAAGAAGCTCGAGAAG |  |
| ScN394K-R | CCTTTTGAGCTTCGCGAGGA |  |
| ScN394K-F | TCCTCGCGAAGCTCAAAAGG |  |
| ScAvrll-R1 | GTCGTCGAGCTTGGCCATCT |  |
| spREC-F | AGATGGCCAAGCTCGACGACAGTTTCTTTCATCGACTTGA |  |
| spREC-R | TCGGCAATCTGCTCATGGAGCGAGTCCCCCTGGCCGCTCA |  |
| ScAfIII-F | CTCCATGAGCAGATTGCCGA |  |
| SpcRN+-F | AGATGGCCAAGCTCGACGACAGCTTCTTCCACAGGCTCGA |  |
| SpcRN++-R | GAGCTTGCCGGAGCGCTTTCTGAGCTTCTTGCCGCCATCGATGTAGCCGG |  |
| SpcRN++-F | AAGAAGCTCAGAAAGCGCTCCGGCAAGCTCGCTTCTCAAGAGGAGTTCTA |  |
| M13-F | TGTAAAACGACGGCCAGTG | Constructed the ABE8e-Sc-sg vector |
| SpR-R | TGAGACCGGCTTATTATGCACG |  |
| SpR-Sc-sg-F | CGTGCATAATAAGCCGGTCTCAGTTTTAGAGCTAGGTACC |  |
| Sc-sg-R | CCGGGTCACGCTGCAAAGCTTTACTAAAAAAAAGCACCGACTCGGTGCCACTT |  |
| ubi-A3A/8e-F | TGTTTGGTGTTACTTCTGCAGGCCACCATGGCCCCAAAGAAG | Amplified TadA8e, Sc++ variants and NLS |
| 8e-R | GGAACCACCGGAGGACCCGC |  |
| 8e-Sc-F | GCGGGTCCTCCGGTGGTTCCGAGAAGAAGTACTCTATCGGCCTCGCCATCGGCACC |  |
| Sc-R | ATCGCCGCCGAGTTGTGAGA |  |
| Sc-NLS-F | TCTCACAACTCGGCGGCGATTCCGGCGGCAGCCCAAAGAAG |  |
| NLS-ter-R | GGCGCGCCCACCCTTGAGCTCTCACACCTTGCGCTTCTTC |  |
| A3A-R | AGATTCTGGGGTGGCCGACT | Amplified A3A, Sc++ variants and UGI |
| A3A-Sc-F | AGTCGGCCACCCCAGAATCTGAGAAGAAGTACTCTATCGGCCTCGcCATCGGCACC |  |
| Sc-UGI-F | TCTCACAACTCGGCGGCGATTCCGGCGGCTCCACCAATTT |  |
| UGI-R | GGCGCGCCCACCCTTGAGCTCTCACACCTTCCTTTTCTTC |  |
| HiTom-OsSLR1-F | ggagtgagtacggtgtgcTGGAGATGGCCATGGGGATG | The prime for Hi-Tom sequence |
| HiTom-OsSLR1-R | gagttggatgctggatggTGACAGTGGACGAGGTGGAA |  |
| HiTom-OsSIP1-F | ggagtgagtacggtgtgcGCCATCGCTCAAGGTGGATC |  |
| HiTom-OsSIP1-R | gagttggatgctggatggTCTCACATTGGCAGGGTTCA |  |
| HiTom-OsIPA1-F | ggagtgagtacggtgtgcAGCTTGAGCTTGCTCTGCAG |  |
| HiTom-OsIPA1-R | gagttggatgctggatggGCAGGCAGCATGAGGTTCTT |  |
| HiTom-OsSPP2-F | ggagtgagtacggtgtgcCTGGTATTATCGAAGCTATTG |  |
| HiTom-OsSPP2-R | gagttggatgctggatggAGTACTGTACAACTGAATCTG |  |
| HiTom-OsCCT1-F | ggagtgagtacggtgtgcTCCTACTCCTCGTCGCTCCC |  |
| HiTom-OsCCT1-R | gagttggatgctggatggTCGAAGAGGTCGCCAGAGGA |  |
| HiTom-OsMTN-F | ggagtgagtacggtgtgcTCCAAGCTCCTCATCGTCAT |  |
| HiTom-OsMTN-R | gagttggatgctggatggCGGGAGAACACTACTAACAT |  |
| HiTom-OsWSL9-F | ggagtgagtacggtgtgcGCTCGGCAACGTCGTCTTCC |  |
| HiTom-OsWSL9-R | gagttggatgctggatggGATTAGTAATGCAACGCGTCA |  |
| HiTom-OsELF1-F | ggagtgagtacggtgtgcGGTGATGATGGCGATAAGTCG |  |
| HiTom-OsELF1-R | gagttggatgctggatggCCAAGGATGCCGTCCTTGAG |  |
| HiTom-OsTSV3-F | ggagtgagtacggtgtgcGGTTGCAGACGTTGGTCTTG |  |
| HiTom-OsTSV3-R | gagttggatgctggatggAACGTAGACCTTTCCAAACC |  |
| HiTom-OsNAL1-F | ggagtgagtacggtgtgcAAGGATTGTTGCCAAGGCAC |  |
| HiTom-OsNAL1-R | gagttggatgctggatgCAAGGATTGCAGGGATATCTG |  |
| HiTom-OsACC-F | ggagtgagtacggtgtgcCATGGCTGCAGAGCTACGAG |  |
| HiTom-OsACC-R | gagttggatgctggatggGGTCAAGCCGACTCATGCAA |  |
| HiTom-OsALS-F | ggagtgagtacggtgtgcAAGATGCTCGAGACTCCAGG |  |
| HiTom-OsALS-R | gagttggatgctggatggCATCATAGGCATACCACTCT |  |
| HiTom-OsACC-I1879V-F | ggagtgagtacggtgtgcAGTGGTGAAATTAGGTGGGT |  |
| HiTom-OsACC-I1879V-R | gagttggatgctggatggCAGTGCAGAATAGCCTGTAA |  |
| HiTom-OsACC-D2176G-F | ggagtgagtacggtgtgcGTCTACATTCCCATGGCTGC |  |
| HiTom-OsACC-D2176G-R | gagttggatgctggatggCAGATCAATTAATGTTGGGTC |  |
| HiTom-OsIPA1-off1-F | ggagtgagtacggtgtgcAAGCTCAGCCTGTTCATACG |  |
| HiTom-OsIPA1-off1-R | gagttggatgctggatggGCATTGCGAGTTTAGTGCCA |  |
| HiTom-OsIPA1-off2-F | ggagtgagtacggtgtgcAGGAATCGGTTGTCGTTGCT |  |
| HiTom-OsIPA1-off2-R | gagttggatgctggatggCCATGTCTCCTAGTATCACC |  |
| HiTom-OsWSL9-off1-F | ggagtgagtacggtgtgcCGGGATGAATTTATCAGGAC |  |
| HiTom-OsWSL9-off1-R | gagttggatgctggatggGCTCGTACTGGCAAGCTACC |  |
| HiTom-OsWSL9-off2-F | ggagtgagtacggtgtgcCTCCTCGTCGGAGTCGAGGA |  |
| HiTom-OsWSL9-off2-R | gagttggatgctggatggGACGATCAAGTCATCCTCGA |  |
| HiTom-OsWSL9-off3-F | ggagtgagtacggtgtgcACATTGCATCCATCCAAGAT |  |
| HiTom-OsWSL9-off3-R | gagttggatgctggatggAACGGCAAGACGCACTTCGA |  |
| HiTom-OsWSL9-off4-F | ggagtgagtacggtgtgcTGGAGCTCGTACTGGCGAGC |  |
| HiTom-OsWSL9-off4-R | gagttggatgctggatggTCGTCGGAGTCGAGGGCGAA |  |
| HiTom-OsWSL9-off5-F | ggagtgagtacggtgtgcTCCTCCTCCTCCACGGCTTC |  |
| HiTom-OsWSL9-off5-R | gagttggatgctggatggCATCCTGTAGCCCACGAATC |  |
| HiTom-OsSPP2-off1-F | ggagtgagtacggtgtgcTGCTGCACTTACCTGTTG |  |
| HiTom-OsSPP2-off1-R | gagttggatgctggatggCTTTCTAAGTTGCTGGTAGG |  |
| HiTom-OsSPP2-off2-F | ggagtgagtacggtgtgcGCTTTGCTTGATAACTATGC |  |
| HiTom-OsSPP2-off2-R | gagttggatgctggatggTCAGTTGTATAGAACGTACG |  |
| OsHOS66-F | TTGGAGGAAGGGACAGGG | 1st round PCR for deep sequencing |
| OsHOS66-R | GCCGCATCAAACATGACACC |  |
| OsGAPDH-F | GATGGAGACGGCGAAGGT |  |
| OsGAPDH-R | TCTGCCAGTCAACAAAGG |  |
| OsROC4-F | CGAAACGAACAGCAAACC |  |
| OsROC4-R | ATGCCTCTGATACATACCCT |  |
| OsWSL9-F | CCGAGGAGATAGGGAGATAAT |  |
| OsWSL9-R | AGACGCATGTACCTGCAAAA |  |
| OsADH1-F | TTTACCATTTCGTCGGGACT |  |
| OsADH1-R | AACGCTGCGGTCAACTCC |  |
| OsHDS-F | AAGACCAGGCTTATTGATTG |  |
| OsHDS-R | TTTCTCCTCCTTGTCTCATAT |  |
| OsIPA1-F | CATACAGCGGCCAAGGTA |  |
| OsIPA1-R | AACATATCGAGCAGAAGTCC |  |
| OsSPP2-F | TCTAACAAAGTTCATGCCAGAT |  |
| OsSPP2-R | AGAACATTCAAGCCCAGAAG |  |
| OsBADH2-F | TGTGGTATTCCGTTGTTATG |  |
| OsBADH2-R | ACTACACCGATAGGCTCTTT |  |
| OsEPSPS-F | TGCCATATTCCTGCCAAGC |  |
| OsEPSPS-R | ACAGCACAGCAAGCCACA |  |
| OsHPPD-F | ACGAGTTCGCCGAGTTCA |  |
| OsHPPD-R | GCTTTATTCCCTTTCTAGTGTC |  |
| OsCPK6-F | GCCAAGGTTGGAAGGAGC |  |
| OsCPK6-R | CCAGGTGGTGCATTATCTGTAT |  |
| OsTSV3-F | TTGTTTCACCGATAGCCT |  |
| OsTSV3-R | CACCCTCAATGAGACCTG |  |
| OsTGW2-F | ACCAGGTCTTGCCTTCCG |  |
| OsTGW2-R | CGTTCGCCATCTTCGTCTA |  |
| OsSULTR-F | AAAGTTGTTCTGGGTGTCG |  |
| OsSULTR-R | TCGTGAGGTAGCAGGAGG |  |
| OsLRR-F | GCAACAACACGCTCCAGG |  |
| OsLRR-R | AATGAAAGCAGGCACCAC |  |
| OsFBK1-F | CTTCTTGCGGATTAGTTT |  |
| OsFBK1-R | CTACCTCTTGCCATTGTT |  |
| OsSLR1-F | TCCCACCTCACCTCGCATTG |  |
| OsSLR1-R | CATCCGCTTGGTGTCCCTC |  |
| OsSIP1-F | GTTCCTGTGGGTGCTGTGCG |  |
| OsSIP1-F | TAGCCTGACGGCGTGTTTGA |  |
| OsGS1-F | TGGTATCGGTGCTGACAAGT |  |
| OsGS1-R | TGAGCTTCTCAATGGCGGAC |  |
| OsACC-D2176G-F | GGCACTCCTTTAGTTCAA |  |
| OsACC-D2176G-R | AGAATAATAGGCTGGTCAA |  |
| OsIPA1-T2-F | AGGGTTCCAAGCAGCGTAAGGA |  |
| OsIPA1-T2-R | TGGTGCTGGGGCTGGACCGTTC |  |
| OsALS-F | AAGGTGAGGCAATCATCGCT |  |
| OsALS-R | CCATGCCAAGCACATCAAAC |  |
| OsMTN-F | CCTCTATCTGACGACCCACA |  |
| OsMTN-R | TAAACGAAGGTTGCCACTAA |  |
| OsTubA2-F | CCTGCATCTTTCCTTGCAGC |  |
| OsTubA2-R | TGGTAGTTGATGCCGCACTT |  |
| OsACC-T2-F | CTCGCTAACTGGAGAGGCTT |  |
| OsACC-T2-R | ATCCTCAGAGATCCTCCTCCG |  |
| OsACC-T3-F | GGCACTCCTTTAGTTCAA |  |
| OsACC-T3-R | AGAATAATAGGCTGGTCAA |  |
| OsCDT1-F | AAAGAGCCTACCTTCATTGCC |  |
| OsCDT1-R | GCACGGAGCGTCGTAAAA |  |
| OsTAC1-F | CACTCCATATTGGGAACACGTT |  |
| OsTAC1-R | GGCAGGACTATTCTTCATCACT |  |
| OsAAT-F | AGGTTAAGTACGCTGGTGCGC |  |
| OsAAT-R | GACGATTCAAAGCAAGAATGGTGCC |  |
| OsELF1-T1-F | CCTCGGTCATCAGTCATCC |  |
| OsELF1-T1-R | CTCGTCCATCATCTTCCTCA |  |
| OsD2-F | CTCTTCTGTCAGCCACTCTG |  |
| OsD2-R | GCTCTAAGATGCCAACAAGAAAGG |  |
| OsWx-F | AGCCAAGCGACGTGAAGAAG |  |
| OsWx-R | GATCAACCAGAGCCACAACC |  |
| OsEV-F | GAGTAGTGTAGTACTGAAGAAGCACAGC |  |
| OsEV-R | GGGAGATGAGAGAGCTTGTGCC |  |
| OsWx-T2-F | GGGTGAGGCTTTGAACCCAG |  |
| OsWx-T2-R | GGCGTGCCGACGACCTTGAT |  |
| OsHOS66-F0 | CGATGTCGATGGATATGGGCTCGGAC | 2nd round PCR for deep sequencing |
| OsHOS66-R0 | TGACCAAGACGAATGTAGGTGCATGC |  |
| OsHOS66-F1 | ACAGTGCGATGGATATGGGCTCGGAC |  |
| OsHOS66-R1 | GCCAATAGACGAATGTAGGTGCATGC |  |
| OsHOS66-F2 | CAGATCCGATGGATATGGGCTCGGAC |  |
| OsHOS66-R2 | CTTGTAAGACGAATGTAGGTGCATGC |  |
| OsGAPDH-F0 | CGATGTTGACAGGTGGTGAGGTCAGG |  |
| OsGAPDH-R0 | TGACCACTGAAATACTACAAGCTTCT |  |
| OsGAPDH-F1 | ACAGTGTGACAGGTGGTGAGGTCAGG |  |
| OsGAPDH-R1 | GCCAATCTGAAATACTACAAGCTTCT |  |
| OsGAPDH-F2 | CAGATCTGACAGGTGGTGAGGTCAGG |  |
| OsGAPDH-R2 | CTTGTACTGAAATACTACAAGCTTCT |  |
| OsROC4-F0 | CGATGTTCGGCAATGGCTGGCATTGA |  |
| OsROC4-R0 | TGACCAATGCCTCTGATACATACCCT |  |
| OsROC4-F1 | ACAGTGTCGGCAATGGCTGGCATTGA |  |
| OsROC4-R1 | GCCAATATGCCTCTGATACATACCCT |  |
| OsROC4-F2 | CAGATCTCGGCAATGGCTGGCATTGA |  |
| OsROC4-R2 | CTTGTAATGCCTCTGATACATACCCT |  |
| OsADH1-F0 | CGATGTTTGGTCTAGGAGCTGTAGGC |  |
| OsADH1-R0 | TGACCACTTCAAATCTGTTGGCGTTC |  |
| OsADH1-F1 | ACAGTGTTGGTCTAGGAGCTGTAGGC |  |
| OsADH1-R1 | GCCAATCTTCAAATCTGTTGGCGTTC |  |
| OsADH1-F2 | CAGATCTTGGTCTAGGAGCTGTAGGC |  |
| OsADH1-R2 | CTTGTACTTCAAATCTGTTGGCGTTC |  |
| OsHDS-F0 | CGATGTTGATGGTGGCATCGGCTTCA |  |
| OsHDS-R0 | TGACCACAACAGCCGGCGAGTTATAC |  |
| OsHDS-F1 | CAGATCTGATGGTGGCATCGGCTTCA |  |
| OsHDS-R1 | CTTGTACAACAGCCGGCGAGTTATAC |  |
| OsHDS-F2 | ACTTGATGATGGTGGCATCGGCTTCA |  |
| OsHDS-R2 | GATCAGCAACAGCCGGCGAGTTATAC |  |
| OsEPSPS-F0 | CGATGTGACATTGAGATTGATGGAGC |  |
| OsEPSPS-R0 | TGACCAAGTGATTGCAGCACCAGCCA |  |
| OsEPSPS-F1 | ACAGTGGACATTGAGATTGATGGAGC |  |
| OsEPSPS-R1 | GCCAATAGTGATTGCAGCACCAGCCA |  |
| OsEPSPS-F2 | CAGATCGACATTGAGATTGATGGAGC |  |
| OsEPSPS-R2 | CTTGTAAGTGATTGCAGCACCAGCCA |  |
| OsHPPD-F0 | CGATGTGAGAGCGGCCTCAACTCGGT |  |
| OsHPPD-R0 | TGACCAATCTCCCTCAGCGTCCCGAG |  |
| OsHPPD-F1 | ACAGTGGAGAGCGGCCTCAACTCGGT |  |
| OsHPPD-R1 | GCCAATATCTCCCTCAGCGTCCCGAG |  |
| OsHPPD-F2 | CAGATCGAGAGCGGCCTCAACTCGGT |  |
| OsHPPD-R2 | CTTGTAATCTCCCTCAGCGTCCCGAG |  |
| OsSULTR-F0 | CGATGTATGCCAAGACGATTCAAGCT |  |
| OsSULTR-R0 | TGACCACGTACTGCCAGTGCTAGGAT |  |
| OsSULTR-F1 | ACAGTGATGCCAAGACGATTCAAGCT |  |
| OsSULTR-R1 | GCCAATCGTACTGCCAGTGCTAGGAT |  |
| OsSULTR-F2 | CAGATCATGCCAAGACGATTCAAGCT |  |
| OsSULTR-R2 | CTTGTACGTACTGCCAGTGCTAGGAT |  |
| OsLRR-F0 | CGATGTTTCCAGAGCCGATCATGAAC |  |
| OsLRR-R0 | TGACCAAATGAAAGCAGGCACCACCC |  |
| OsLRR-F1 | ACAGTGTTCCAGAGCCGATCATGAAC |  |
| OsLRR-R1 | GCCAATAATGAAAGCAGGCACCACCC |  |
| OsLRR-F2 | CAGATCTTCCAGAGCCGATCATGAAC |  |
| OsLRR-R2 | CTTGTAAATGAAAGCAGGCACCACCC |  |
| OsFBK1-F0 | CGATGTATTACACCATTACATTGGC |  |
| OsFBK1-R0 | TGACCACCAAGAATGCCTGTGGAGTG |  |
| OsFBK1-F1 | ACAGTGATTACACCATTACATTGGC |  |
| OsFBK1-R1 | GCCAATCCAAGAATGCCTGTGGAGTG |  |
| OsFBK1-F2 | CAGATCATTACACCATTACATTGGC |  |
| OsFBK1-R2 | CTTGTACCAAGAATGCCTGTGGAGTG |  |
| OsGAPDH-T2-F0 | CGATGTGATGGAGACGGCGAAGGTGG |  |
| OsGAPDH-T2-R0 | TGACCATCAGTTAGCTTCGCTTCAGA |  |
| OsGAPDH-T2-F1 | ACAGTGGATGGAGACGGCGAAGGTGG |  |
| OsGAPDH-T2-R1 | GCCAATTCAGTTAGCTTCGCTTCAGA |  |
| OsGAPDH-T2-F2 | CAGATCGATGGAGACGGCGAAGGTGG |  |
| OsGAPDH-T2-R2 | CTTGTATCAGTTAGCTTCGCTTCAGA |  |
| OsGAPDH-T2-F3 | ATCACGGATGGAGACGGCGAAGGTGG |  |
| OsGAPDH-T2-R3 | TTAGGCTCAGTTAGCTTCGCTTCAGA |  |
| OsGAPDH-T2-F4 | ACTTGAGATGGAGACGGCGAAGGTGG |  |
| OsGAPDH-T2-R4 | GATCAGTCAGTTAGCTTCGCTTCAGA |  |
| OsGAPDH-T2-F5 | TAGCTTGATGGAGACGGCGAAGGTGG |  |
| OsGAPDH-T2-R5 | GGCTACTCAGTTAGCTTCGCTTCAGA |  |
| OsWSL9-F0 | ACAGTGCTACGTGGTGAAGCAGCGGT |  |
| OsWSL9-R0 | GCCAATGGCTTTCACATCATCAATTCAT |  |
| OsWSL9-F1 | ATCACGCTACGTGGTGAAGCAGCGGT |  |
| OsWSL9-R1 | TTAGGCGGCTTTCACATCATCAATTCAT |  |
| OsWSL9-F2 | TAGCTTCTACGTGGTGAAGCAGCGGT |  |
| OsWSL9-R2 | GGCTACGGCTTTCACATCATCAATTCAT |  |
| OsWSL9-F3 | ATGTCACTACGTGGTGAAGCAGCGGT |  |
| OsWSL9-R3 | CCGTCCGGCTTTCACATCATCAATTCAT |  |
| OsWSL9-F4 | GTGAAACTACGTGGTGAAGCAGCGGT |  |
| OsWSL9-R4 | GTGGCCGGCTTTCACATCATCAATTCAT |  |
| OsWSL9-F5 | GTTTCGCTACGTGGTGAAGCAGCGGT |  |
| OsWSL9-R5 | CGTACGGGCTTTCACATCATCAATTCAT |  |
| OsIPA1-F0 | GTGGCCCTGGTCCTAGCCATCATGCC |  |
| OsIPA1-R0 | GTTTCGGCAGGCAGCATGAGGTTCTT |  |
| OsIPA1-F1 | CGATGTCTGGTCCTAGCCATCATGCC |  |
| OsIPA1-R1 | TGACCAGCAGGCAGCATGAGGTTCTT |  |
| OsIPA1-F2 | CAGATCCTGGTCCTAGCCATCATGCC |  |
| OsIPA1-R2 | CTTGTAGCAGGCAGCATGAGGTTCTT |  |
| OsIPA1-F3 | ATCACGCTGGTCCTAGCCATCATGCC |  |
| OsIPA1-R3 | TTAGGCGCAGGCAGCATGAGGTTCTT |  |
| OsIPA1-F4 | GTGAAACTGGTCCTAGCCATCATGCC |  |
| OsIPA1-R4 | AGTCAAGCAGGCAGCATGAGGTTCTT |  |
| OsIPA1-F5 | AGTTCCCTGGTCCTAGCCATCATGCC |  |
| OsIPA1-R5 | GTGGCCGCAGGCAGCATGAGGTTCTT |  |
| OsSPP2-F1 | ACTGATCTGGTATTATCGAAGCTATTG |  |
| OsSPP2-R1 | CATGGCAGTACTGTACAACTGAATCTG |  |
| OsSPP2-F2 | GTGAAACTGGTATTATCGAAGCTATTG |  |
| OsSPP2-R2 | CAAAAGAGTACTGTACAACTGAATCTG |  |
| OsSPP2-F3 | CGTACGCTGGTATTATCGAAGCTATTG |  |
| OsSPP2-R3 | CACGATAGTACTGTACAACTGAATCTG |  |
| OsSPP2-F4 | CTATACCTGGTATTATCGAAGCTATTG |  |
| OsSPP2-R4 | CTCAGAAGTACTGTACAACTGAATCTG |  |
| OsSPP2-F5 | GTAGAGCTGGTATTATCGAAGCTATTG |  |
| OsSPP2-R5 | GTCCGCAGTACTGTACAACTGAATCTG |  |
| OsBADH2-F0 | CGTACGTCATGGCATATGCGAGCATT |  |
| OsBADH2-R0 | GAGTGGCATCAGGATTCATGTTCAGG |  |
| OsBADH2-F1 | ATCACGTCATGGCATATGCGAGCATT |  |
| OsBADH2-R1 | TTAGGCCATCAGGATTCATGTTCAGG |  |
| OsBADH2-F2 | CGATGTTCATGGCATATGCGAGCATT |  |
| OsBADH2-R2 | TGACCACATCAGGATTCATGTTCAGG |  |
| OsBADH2-F3 | ACAGTGTCATGGCATATGCGAGCATT |  |
| OsBADH2-R3 | GCCAATCATCAGGATTCATGTTCAGG |  |
| OsBADH2-F4 | TAGCTTTCATGGCATATGCGAGCATT |  |
| OsBADH2-R4 | AGTTCCCATCAGGATTCATGTTCAGG |  |
| OsBADH2-F5 | ACTTGATCATGGCATATGCGAGCATT |  |
| OsBADH2-R5 | GATCAGCATCAGGATTCATGTTCAGG |  |
| OsCPK6-F0 | GTAGCAGAGAGAGGATCGTCGTCGA |  |
| OsCPK6-R0 | ACTGATTACTGCAACTGCTTGGATAC |  |
| OsCPK6-F1 | ACAGTGAGAGAGAGGATCGTCGTCGA |  |
| OsCPK6-R1 | GCCAATTACTGCAACTGCTTGGATAC |  |
| OsCPK6-F2 | CAGATCAGAGAGAGGATCGTCGTCGA |  |
| OsCPK6-R2 | CTTGTATACTGCAACTGCTTGGATAC |  |
| OsCPK6-F3 | ATCACGAGAGAGAGGATCGTCGTCGA |  |
| OsCPK6-R3 | TTAGGCTACTGCAACTGCTTGGATAC |  |
| OsCPK6-F4 | GTGAAAAGAGAGAGGATCGTCGTCGA |  |
| OsCPK6-R4 | GTGGCCTACTGCAACTGCTTGGATAC |  |
| OsCPK6-F5 | TAGCTTAGAGAGAGGATCGTCGTCGA |  |
| OsCPK6-R5 | GGCTACTACTGCAACTGCTTGGATAC |  |
| OsTSV-F0 | CGATGTGGTGTTAATTCATGGTCAGC |  |
| OsTSV-R0 | TGACCATCACAACGTAGACCTTTCCA |  |
| OsTSV-F1 | CAGATCGGTGTTAATTCATGGTCAGC |  |
| OsTSV-R1 | CTTGTATCACAACGTAGACCTTTCCA |  |
| OsTSV-F2 | ACTTGAGGTGTTAATTCATGGTCAGC |  |
| OsTSV-R2 | GATCAGTCACAACGTAGACCTTTCCA |  |
| OsTSV-F3 | AGTCAAGGTGTTAATTCATGGTCAGC |  |
| OsTSV-R3 | AGTTCCTCACAACGTAGACCTTTCCA |  |
| OsTSV-F4 | GTGAAAGGTGTTAATTCATGGTCAGC |  |
| OsTSV-R4 | GTGGCCTCACAACGTAGACCTTTCCA |  |
| OsTSV-F5 | GTTTCGGGTGTTAATTCATGGTCAGC |  |
| OsTSV-R5 | CGTACGTCACAACGTAGACCTTTCCA |  |
| OsTGW2-F0 | CGATGTGTGTGAAACTGTGATCGATT |  |
| OsTGW2-R0 | TGACCACAGTGCTTGCACCTACCGAT |  |
| OsTGW2-F1 | ACAGTGGTGTGAAACTGTGATCGATT |  |
| OsTGW2-R1 | GCCAATCAGTGCTTGCACCTACCGAT |  |
| OsTGW2-F2 | CAGATCGTGTGAAACTGTGATCGATT |  |
| OsTGW2-R2 | CTTGTACAGTGCTTGCACCTACCGAT |  |
| OsTGW2-F3 | ATCACGGTGTGAAACTGTGATCGATT |  |
| OsTGW2-R3 | TTAGGCCAGTGCTTGCACCTACCGAT |  |
| OsTGW2-F4 | ACTTGAGTGTGAAACTGTGATCGATT |  |
| OsTGW2-R4 | GATCAGCAGTGCTTGCACCTACCGAT |  |
| OsTGW2-F5 | TAGCTTGTGTGAAACTGTGATCGATT |  |
| OsTGW2-R5 | GGCTACCAGTGCTTGCACCTACCGAT |  |
| OsSLR1-F0 | ATGAGCTGGAGATGGCCATGGGGAT |  |
| OsSLR1-R0 | ATTCCTTGACAGTGGACGAGGTGGAA |  |
| OsSLR1-F1 | CAGATCTGGAGATGGCCATGGGGAT |  |
| OsSLR1-R1 | CTTGTATGACAGTGGACGAGGTGGAA |  |
| OsSLR1-F2 | ACTTGATGGAGATGGCCATGGGGAT |  |
| OsSLR1-R2 | GATCAGTGACAGTGGACGAGGTGGAA |  |
| OsSLR1-F3 | AGTCAATGGAGATGGCCATGGGGAT |  |
| OsSLR1-R3 | AGTTCCTGACAGTGGACGAGGTGGAA |  |
| OsSLR1-F4 | GTGAAATGGAGATGGCCATGGGGAT |  |
| OsSLR1-R4 | GTGGCCTGACAGTGGACGAGGTGGAA |  |
| OsSLR1-F5 | TAGCTTTGGAGATGGCCATGGGGAT |  |
| OsSLR1-R5 | GGCTACTGACAGTGGACGAGGTGGAA |  |
| OsSIP-F0 | CGATGTCGATCTCGGAGCTGATGCCG |  |
| OsSIP-R0 | TGACCATCTCACATTGGCAGGGTTCA |  |
| OsSIP-F1 | CAGATCCGATCTCGGAGCTGATGCCG |  |
| OsSIP-R1 | CTTGTATCTCACATTGGCAGGGTTCA |  |
| OsSIP-F2 | ACTTGACGATCTCGGAGCTGATGCCG |  |
| OsSIP-R2 | GATCAGTCTCACATTGGCAGGGTTCA |  |
| OsSIP-F3 | AGTCAACGATCTCGGAGCTGATGCCG |  |
| OsSIP-R3 | AGTTCCTCTCACATTGGCAGGGTTCA |  |
| OsSIP-F4 | GTGAAACGATCTCGGAGCTGATGCCG |  |
| OsSIP-R4 | GTGGCCTCTCACATTGGCAGGGTTCA |  |
| OsSIP-F5 | TAGCTTCGATCTCGGAGCTGATGCCG |  |
| OsSIP-R5 | GGCTACTCTCACATTGGCAGGGTTCA |  |
| OsGS1-F0 | ACTTGATCAGAACGGTCTGATCGAAT |  |
| OsGS1-R0 | GATCAGCTCATCGACTTGGTGCTGAA |  |
| OsGS1-F1 | CGATGTTCAGAACGGTCTGATCGAAT |  |
| OsGS1-R1 | TGACCACTCATCGACTTGGTGCTGAA |  |
| OsGS1-F2 | ACAGTGTCAGAACGGTCTGATCGAAT |  |
| OsGS1-R2 | GCCAATCTCATCGACTTGGTGCTGAA |  |
| OsGS1-F3 | CAGATCTCAGAACGGTCTGATCGAAT |  |
| OsGS1-R3 | CTTGTACTCATCGACTTGGTGCTGAA |  |
| OsGS1-F4 | ATCACGTCAGAACGGTCTGATCGAAT |  |
| OsGS1-R4 | TTAGGCCTCATCGACTTGGTGCTGAA |  |
| OsACC-D2176G-F0 | ACTTGAAGGACATACAATCAGCCTGC |  |
| OsACC-D2176G-R0 | GATCAGTCCTGGAGTTCCTCTGACCT |  |
| OsACC-D2176G-F1 | CGATGTAGGACATACAATCAGCCTGC |  |
| OsACC-D2176G-R1 | TGACCATCCTGGAGTTCCTCTGACCT |  |
| OsACC-D2176G-F2 | ACAGTGAGGACATACAATCAGCCTGC |  |
| OsACC-D2176G-R2 | GCCAATTCCTGGAGTTCCTCTGACCT |  |
| OsACC-D2176G-F3 | CAGATCAGGACATACAATCAGCCTGC |  |
| OsACC-D2176G-R3 | CTTGTATCCTGGAGTTCCTCTGACCT |  |
| OsACC-D2176G-F4 | ATCACGAGGACATACAATCAGCCTGC |  |
| OsACC-D2176G-R4 | TTAGGCTCCTGGAGTTCCTCTGACCT |  |
| OsIPA1-T2-F0 | ACTTGATATGGTGCCAACACATACAG |  |
| OsIPA1-T2-R0 | GATCAGTGCCACAGGATTGCCATCAA |  |
| OsIPA1-T2-F1 | CGATGTTATGGTGCCAACACATACAG |  |
| OsIPA1-T2-R1 | TGACCATGCCACAGGATTGCCATCAA |  |
| OsIPA1-T2-F2 | ACAGTGTATGGTGCCAACACATACAG |  |
| OsIPA1-T2-R2 | GCCAATTGCCACAGGATTGCCATCAA |  |
| OsIPA1-T2-F3 | CAGATCTATGGTGCCAACACATACAG |  |
| OsIPA1-T2-R3 | CTTGTATGCCACAGGATTGCCATCAA |  |
| OsIPA1-T2-F4 | ATCACGTATGGTGCCAACACATACAG |  |
| OsIPA1-T2-R4 | TTAGGCTGCCACAGGATTGCCATCAA |  |
| OsALS-T2-F0 | ACTTGAGCCATCAAGAAGATGCTCGA |  |
| OsALS-T2-R0 | GATCAGCATCATAGGCATACCACTCT |  |
| OsALS-T2-F1 | CGATGTGCCATCAAGAAGATGCTCGA |  |
| OsALS-T2-R1 | TGACCACATCATAGGCATACCACTCT |  |
| OsALS-T2-F2 | ACAGTGGCCATCAAGAAGATGCTCGA |  |
| OsALS-T2-R2 | GCCAATCATCATAGGCATACCACTCT |  |
| OsALS-T2-F3 | CAGATCGCCATCAAGAAGATGCTCGA |  |
| OsALS-T2-R3 | CTTGTACATCATAGGCATACCACTCT |  |
| OsALS-T2-F4 | ATCACGGCCATCAAGAAGATGCTCGA |  |
| OsALS-T2-R4 | TTAGGCCATCATAGGCATACCACTCT |  |
| OsMTN-F0 | CGATGTTCCAAGCTCCTCATCGTCAT |  |
| OsMTN-R0 | TGACCACCAACAGCAGTAAGTGCAGC |  |
| OsMTN-F1 | CAGATCTCCAAGCTCCTCATCGTCAT |  |
| OsMTN-R1 | CTTGTACCAACAGCAGTAAGTGCAGC |  |
| OsMTN-F2 | ACTTGATCCAAGCTCCTCATCGTCAT |  |
| OsMTN-R2 | GATCAGCCAACAGCAGTAAGTGCAGC |  |
| OsMTN-F3 | AGTCAATCCAAGCTCCTCATCGTCAT |  |
| OsMTN-R3 | AGTTCCCCAACAGCAGTAAGTGCAGC |  |
| OsMTN-F4 | GTGAAATCCAAGCTCCTCATCGTCAT |  |
| OsMTN-R4 | GTGGCCCCAACAGCAGTAAGTGCAGC |  |
| OsTubA2-F0 | ACTTGATGTGCTGCAGGTCATCTCCT |  |
| OsTubA2-R0 | GATCAGAAGGCGCTGTTGGTGATCTC |  |
| OsTubA2-F1 | CGATGTTGTGCTGCAGGTCATCTCCT |  |
| OsTubA2-R1 | TGACCAAAGGCGCTGTTGGTGATCTC |  |
| OsTubA2-F2 | ACAGTGTGTGCTGCAGGTCATCTCCT |  |
| OsTubA2-R2 | GCCAATAAGGCGCTGTTGGTGATCTC |  |
| OsTubA2-F3 | CAGATCTGTGCTGCAGGTCATCTCCT |  |
| OsTubA2-R3 | CTTGTAAAGGCGCTGTTGGTGATCTC |  |
| OsTubA2-F4 | ATCACGTGTGCTGCAGGTCATCTCCT |  |
| OsTubA2-R4 | TTAGGCAAGGCGCTGTTGGTGATCTC |  |
| OsACC-T2-F0 | ACTTGATAGGACATACAATCAGCCTG |  |
| OsACC-T2-R0 | GATCAGCCGACTCATGCAATCCTGGA |  |
| OsACC-T2-F1 | CGATGTTAGGACATACAATCAGCCTG |  |
| OsACC-T2-R1 | TGACCACCGACTCATGCAATCCTGGA |  |
| OsACC-T2-F2 | ACAGTGTAGGACATACAATCAGCCTG |  |
| OsACC-T2-R2 | GCCAATCCGACTCATGCAATCCTGGA |  |
| OsACC-T2-F3 | CAGATCTAGGACATACAATCAGCCTG |  |
| OsACC-T2-R3 | CTTGTACCGACTCATGCAATCCTGGA |  |
| OsACC-T2-F4 | ATCACGTAGGACATACAATCAGCCTG |  |
| OsACC-T2-R4 | TTAGGCCCGACTCATGCAATCCTGGA |  |
| OsACC-T3-R0 | ACTTGACATAGCACATAAGATGCAGC |  |
| OsACC-T3-F1 | GATCAGCCAACAGTTCTTCCAGTCAC |  |
| OsACC-T3-R1 | CGATGTCATAGCACATAAGATGCAGC |  |
| OsACC-T3-F2 | TGACCACCAACAGTTCTTCCAGTCAC |  |
| OsACC-T3-R2 | ACAGTGCATAGCACATAAGATGCAGC |  |
| OsACC-T3-F3 | GCCAATCCAACAGTTCTTCCAGTCAC |  |
| OsACC-T3-R3 | CAGATCCATAGCACATAAGATGCAGC |  |
| OsACC-T3-F4 | CTTGTACCAACAGTTCTTCCAGTCAC |  |
| OsACC-T3-R4 | ATCACGCATAGCACATAAGATGCAGC |  |
| OsACC-T3-R0 | TTAGGCCCAACAGTTCTTCCAGTCAC |  |
| OsWx-F0 | CGATGTCGTCTTGTTCAGAAGTTCAG |  |
| OsWx-R0 | TGACCAAGATCTCAGGCTCTTCAAGG |  |
| OsWx-F1 | ACAGTGCGTCTTGTTCAGAAGTTCAG |  |
| OsWx-R1 | GCCAATAGATCTCAGGCTCTTCAAGG |  |
| OsWx-F2 | CAGATCCGTCTTGTTCAGAAGTTCAG |  |
| OsWx-R2 | CTTGTAAGATCTCAGGCTCTTCAAGG |  |
| OsWx-F3 | ACTTGACGTCTTGTTCAGAAGTTCAG |  |
| OsWx-R3 | GATCAGAGATCTCAGGCTCTTCAAGG |  |
| OsWx-F4 | ATCACGCGTCTTGTTCAGAAGTTCAG |  |
| OsWx-R4 | TTAGGCAGATCTCAGGCTCTTCAAGG |  |
| OsWx-T2-F0 | CGATGTAGCTGCTCAAGAGCATGGAG |  |
| OsWx-T2-R0 | TGACCATACCGTTCCGTATCTCATCC |  |
| OsWx-T2-F1 | ACAGTGAGCTGCTCAAGAGCATGGAG |  |
| OsWx-T2-R1 | GCCAATTACCGTTCCGTATCTCATCC |  |
| OsWx-T2-F2 | CAGATCAGCTGCTCAAGAGCATGGAG |  |
| OsWx-T2-R2 | CTTGTATACCGTTCCGTATCTCATCC |  |
| OsWx-T2-F3 | ATCACGAGCTGCTCAAGAGCATGGAG |  |
| OsWx-T2-R3 | TTAGGCTACCGTTCCGTATCTCATCC |  |
| OsWx-T2-F4 | ACTTGAAGCTGCTCAAGAGCATGGAG |  |
| OsWx-T2-R4 | TAGCTTTACCGTTCCGTATCTCATCC |  |
| OsCDT1-F0 | ATCACGATGGGCCTCATCGCCATCTA |  |
| OsCDT1-R0 | TTAGGCACAATCAATCAGTGGTCGCC |  |
| OsCDT1-F1 | TGACCAATGGGCCTCATCGCCATCTA |  |
| OsCDT1-R1 | CGATGTACAATCAATCAGTGGTCGCC |  |
| OsCDT1-F2 | ACAGTGATGGGCCTCATCGCCATCTA |  |
| OsCDT1-R2 | GCCAATACAATCAATCAGTGGTCGCC |  |
| OsCDT1-F3 | ACTTGAATGGGCCTCATCGCCATCTA |  |
| OsCDT1-R3 | GATCAGACAATCAATCAGTGGTCGCC |  |
| OsCDT1-F4 | CAGATCATGGGCCTCATCGCCATCTA |  |
| OsCDT1-R4 | CTTGTAACAATCAATCAGTGGTCGCC |  |
| OsAAT-F0 | CGATGTGCTCGACCTGATCGGTGCTC |  |
| OsAAT-R0 | TGACCAATCCACCACCAATCCAATCC |  |
| OsAAT-F1 | ACAGTGGCTCGACCTGATCGGTGCTC |  |
| OsAAT-R1 | GCCAATATCCACCACCAATCCAATCC |  |
| OsAAT-F2 | CAGATCGCTCGACCTGATCGGTGCTC |  |
| OsAAT-R2 | CTTGTAATCCACCACCAATCCAATCC |  |
| OsAAT-F3 | ATCACGGCTCGACCTGATCGGTGCTC |  |
| OsAAT-R3 | TTAGGCATCCACCACCAATCCAATCC |  |
| OsAAT-F4 | ACTTGAGCTCGACCTGATCGGTGCTC |  |
| OsAAT-R4 | GATCAGATCCACCACCAATCCAATCC |  |
| OsEV-F0 | CGATGTGCAGACCAAGATCCCAAGAA |  |
| OsEV-R0 | TGACCATTGCTTTCATTCTTCAGTGC |  |
| OsEV-F1 | ACAGTGGCAGACCAAGATCCCAAGAA |  |
| OsEV-R1 | GCCAATTTGCTTTCATTCTTCAGTGC |  |
| OsEV-F2 | CAGATCGCAGACCAAGATCCCAAGAA |  |
| OsEV-R2 | CTTGTATTGCTTTCATTCTTCAGTGC |  |
| OsEV-F3 | ATCACGGCAGACCAAGATCCCAAGAA |  |
| OsEV-R3 | TTAGGCTTGCTTTCATTCTTCAGTGC |  |
| OsEV-F4 | ACTTGAGCAGACCAAGATCCCAAGAA |  |
| OsEV-R4 | GATCAGTTGCTTTCATTCTTCAGTGC |  |
| OsTAC1-F0 | CGATGTCAATTACTGCTTGAGGCTAC |  |
| OsTAC1-R0 | TGACCAGGCAGGACTATTCTTCATCA |  |
| OsTAC1-F1 | ACAGTGCAATTACTGCTTGAGGCTAC |  |
| OsTAC1-R1 | GCCAATGGCAGGACTATTCTTCATCA |  |
| OsTAC1-F2 | CAGATCCAATTACTGCTTGAGGCTAC |  |
| OsTAC1-R2 | CTTGTAGGCAGGACTATTCTTCATCA |  |
| OsTAC1-F3 | ACTTGACAATTACTGCTTGAGGCTAC |  |
| OsTAC1-R3 | GATCAGGGCAGGACTATTCTTCATCA |  |
| OsTAC1-F4 | ATCACGCAATTACTGCTTGAGGCTAC |  |
| OsTAC1-R4 | TTAGGCGGCAGGACTATTCTTCATCA |  |
| OsD2-F0 | CGATGTATCGACGTGCTCATCGGAGA |  |
| OsD2-R0 | TGACCAGGTATACAGGCTTACTTCCA |  |
| OsD2-F1 | ACAGTGATCGACGTGCTCATCGGAGA |  |
| OsD2-R1 | GCCAATGGTATACAGGCTTACTTCCA |  |
| OsD2-F2 | ACTTGAATCGACGTGCTCATCGGAGA |  |
| OsD2-R2 | GATCAGGGTATACAGGCTTACTTCCA |  |
| OsD2-F3 | ATCACGATCGACGTGCTCATCGGAGA |  |
| OsD2-R3 | TTAGGCGGTATACAGGCTTACTTCCA |  |
| OsD2-F4 | CAGATCATCGACGTGCTCATCGGAGA |  |
| OsD2-R4 | CTTGTAGGTATACAGGCTTACTTCCA |  |
| OsELF1-T2-F0 | CGATGTGAGGCCAGTGCGCTGGTAAA |  |
| OsELF1-T2-R0 | TGACCACACCGGCTGCTTCCCTCCGT |  |
| OsELF1-T2-F1 | CAGATCGAGGCCAGTGCGCTGGTAAA |  |
| OsELF1-T2-R1 | CTTGTACACCGGCTGCTTCCCTCCGT |  |
| OsELF1-T2-F2 | ACTTGAGAGGCCAGTGCGCTGGTAAA |  |
| OsELF1-T2-R2 | GATCAGCACCGGCTGCTTCCCTCCGT |  |
| OsELF1-T2-F3 | AGTCAAGAGGCCAGTGCGCTGGTAAA |  |
| OsELF1-T2-R3 | AGTTCCCACCGGCTGCTTCCCTCCGT |  |
| OsELF1-T2-F4 | GTGAAAGAGGCCAGTGCGCTGGTAAA |  |
| OsELF1-T2-R4 | GTGGCCCACCGGCTGCTTCCCTCCGT |  |
| OsWSL9-mis1-F | CGATGTCTACGTGGTGAAGCAGCGGT | 2nd round mismatch PCR for deep sequencing |
| OsWSL9-mis1-R | TGACCAGGCTTTCACATCATCAATTCAT |  |
| OsWSL9-mis2-F | ACAGTGCTACGTGGTGAAGCAGCGGT |  |
| OsWSL9-mis2-R | GCCAATGGCTTTCACATCATCAATTCAT |  |
| OsWSL9-mis3-F | CAGATCCTACGTGGTGAAGCAGCGGT |  |
| OsWSL9-mis3-R | CTTGTAGGCTTTCACATCATCAATTCAT |  |
| OsWSL9-mis4-F | ATCACGCTACGTGGTGAAGCAGCGGT |  |
| OsWSL9-mis4-R | TTAGGCGGCTTTCACATCATCAATTCAT |  |
| OsWSL9-mis5-F | ACTTGACTACGTGGTGAAGCAGCGGT |  |
| OsWSL9-mis5-R | GATCAGGGCTTTCACATCATCAATTCAT |  |
| OsWSL9-mis6-F | TAGCTTCTACGTGGTGAAGCAGCGGT |  |
| OsWSL9-mis6-R | GGCTACGGCTTTCACATCATCAATTCAT |  |
| OsWSL9-mis7-F | AGTCAACTACGTGGTGAAGCAGCGGT |  |
| OsWSL9-mis7-R | AGTTCCGGCTTTCACATCATCAATTCAT |  |
| OsWSL9-mis8-F | ATGTCACTACGTGGTGAAGCAGCGGT |  |
| OsWSL9-mis8-R | CCGTCCGGCTTTCACATCATCAATTCAT |  |
| OsWSL9-mis9-F | GTAGAGCTACGTGGTGAAGCAGCGGT |  |
| OsWSL9-mis9-R | GTCCGCGGCTTTCACATCATCAATTCAT |  |
| OsWSL9-mis10-F | GTGAAACTACGTGGTGAAGCAGCGGT |  |
| OsWSL9-mis10-R | GTGGCCGGCTTTCACATCATCAATTCAT |  |
| OsWSL9-mis11-F | GTTTCGCTACGTGGTGAAGCAGCGGT |  |
| OsWSL9-mis11-R | CGTACGGGCTTTCACATCATCAATTCAT |  |
| OsWSL9-mis12-F | GAGTGGCTACGTGGTGAAGCAGCGGT |  |
| OsWSL9-mis12-R | GGTAGCGGCTTTCACATCATCAATTCAT |  |
| OsWSL9-mis13-F | ACTGATCTACGTGGTGAAGCAGCGGT |  |
| OsWSL9-mis13-R | ATGAGCGGCTTTCACATCATCAATTCAT |  |
| OsWSL9-mis14-F | ATTCCTCTACGTGGTGAAGCAGCGGT |  |
| OsWSL9-mis14-R | CAAAAGGGCTTTCACATCATCAATTCAT |  |
| OsWSL9-mis15-F | CAACTACTACGTGGTGAAGCAGCGGT |  |
| OsWSL9-mis15-R | CACCGGGGCTTTCACATCATCAATTCAT |  |
| OsWSL9-mis16-F | CACGATCTACGTGGTGAAGCAGCGGT |  |
| OsWSL9-mis16-R | CACTCAGGCTTTCACATCATCAATTCAT |  |
| OsWSL9-mis17-F | CAGGCGCTACGTGGTGAAGCAGCGGT |  |
| OsWSL9-mis17-R | CATGGCGGCTTTCACATCATCAATTCAT |  |
| OsWSL9-mis18-F | CATTTTCTACGTGGTGAAGCAGCGGT |  |
| OsWSL9-mis18-R | CCAACAGGCTTTCACATCATCAATTCAT |  |
| OsWSL9-mis19-F | CGGAATCTACGTGGTGAAGCAGCGGT |  |
| OsWSL9-mis19-R | CTAGCTGGCTTTCACATCATCAATTCAT |  |
| OsWSL9-mis20-F | GCGCTACTACGTGGTGAAGCAGCGGT |  |
| OsWSL9-mis20-R | TAATCGGGCTTTCACATCATCAATTCAT |  |
| OsWSL9-mis21-F | TACAGCCTACGTGGTGAAGCAGCGGT |  |
| OsWSL9-mis21-R | TATAATGGCTTTCACATCATCAATTCAT |  |
| OsSLR1-mis1-F | CGATGTTGGAGATGGCCATGGGGAT |  |
| OsSLR1-mis1-R | TGACCATGACAGTGGACGAGGTGGAA |  |
| OsSLR1-mis2-F | ACAGTGTGGAGATGGCCATGGGGAT |  |
| OsSLR1-mis2-R | GCCAATTGACAGTGGACGAGGTGGAA |  |
| OsSLR1-mis3-F | CAGATCTGGAGATGGCCATGGGGAT |  |
| OsSLR1-mis3-R | CTTGTATGACAGTGGACGAGGTGGAA |  |
| OsSLR1-mis4-F | ATCACGTGGAGATGGCCATGGGGAT |  |
| OsSLR1-mis4-R | TTAGGCTGACAGTGGACGAGGTGGAA |  |
| OsSLR1-mis5-F | ACTTGATGGAGATGGCCATGGGGAT |  |
| OsSLR1-mis5-R | GATCAGTGACAGTGGACGAGGTGGAA |  |
| OsSLR1-mis6-F | TAGCTTTGGAGATGGCCATGGGGAT |  |
| OsSLR1-mis6-R | GGCTACTGACAGTGGACGAGGTGGAA |  |
| OsSLR1-mis7-F | AGTCAATGGAGATGGCCATGGGGAT |  |
| OsSLR1-mis7-R | AGTTCCTGACAGTGGACGAGGTGGAA |  |
| OsSLR1-mis8-F | ATGTCATGGAGATGGCCATGGGGAT |  |
| OsSLR1-mis8-R | CCGTCCTGACAGTGGACGAGGTGGAA |  |
| OsSLR1-mis9-F | GTAGAGTGGAGATGGCCATGGGGAT |  |
| OsSLR1-mis9-R | GTCCGCTGACAGTGGACGAGGTGGAA |  |
| OsSLR1-mis10-F | GTGAAATGGAGATGGCCATGGGGAT |  |
| OsSLR1-mis10-R | GTGGCCTGACAGTGGACGAGGTGGAA |  |
| OsSLR1-mis11-F | GTTTCGTGGAGATGGCCATGGGGAT |  |
| OsSLR1-mis11-R | CGTACGTGACAGTGGACGAGGTGGAA |  |
| OsSLR1-mis12-F | GAGTGGTGGAGATGGCCATGGGGAT |  |
| OsSLR1-mis12-R | GGTAGCTGACAGTGGACGAGGTGGAA |  |
| OsSLR1-mis13-F | ACTGATTGGAGATGGCCATGGGGAT |  |
| OsSLR1-mis13-R | ATGAGCTGACAGTGGACGAGGTGGAA |  |
| OsSLR1-mis14-F | ATTCCTTGGAGATGGCCATGGGGAT |  |
| OsSLR1-mis14-R | CAAAAGTGACAGTGGACGAGGTGGAA |  |
| OsSLR1-mis15-F | CAACTATGGAGATGGCCATGGGGAT |  |
| OsSLR1-mis15-R | CACCGGTGACAGTGGACGAGGTGGAA |  |
| OsSLR1-mis16-F | CACGATTGGAGATGGCCATGGGGAT |  |
| OsSLR1-mis16-R | CACTCATGACAGTGGACGAGGTGGAA |  |
| OsSLR1-mis17-F | CAGGCGTGGAGATGGCCATGGGGAT |  |
| OsSLR1-mis17-R | CATGGCTGACAGTGGACGAGGTGGAA |  |
| OsSLR1-mis18-F | CATTTTTGGAGATGGCCATGGGGAT |  |
| OsSLR1-mis18-R | CCAACATGACAGTGGACGAGGTGGAA |  |
| OsSLR1-mis19-F | CGGAATTGGAGATGGCCATGGGGAT |  |
| OsSLR1-mis19-R | CTAGCTTGACAGTGGACGAGGTGGAA |  |
| OsSLR1-mis20-F | GCGCTATGGAGATGGCCATGGGGAT |  |
| OsSLR1-mis20-R | TAATCGTGACAGTGGACGAGGTGGAA |  |
| OsSLR1-mis21-F | TACAGCTGGAGATGGCCATGGGGAT |  |
| OsSLR1-mis21-R | TATAATTGACAGTGGACGAGGTGGAA |  |

**Supplementary sequences.** Complete amino acid sequences of the Sc++, ScRN++, Spc+, SpcRN+ and SpcRN++ and gRNA scaffold sequence in this study. The R221 and N394 amino acids are shown in orange. The R221K and N394K mutations are shown in blue. Ten positively charged amino acids is shown in red. The T1227K mutation is shown in green. The REC region of SpCas9 is shown in gray.

**gRNA scaffold sequence**

GTTTTAGAGCTAGGTACCTAGCAAGTTAAAATAAGGCTAGTCCGTTATCAACTTGAAAAAGTGGCACCGAGTCGGTGC

**Sc++ (1375 aa)**

MEKKYSIGLDIGTNSVGWAVITDDYKVPSKKFKVLGNTNRKSIKKNLMGALLFDSGETAEATRLKRTARRRYTRRKNRIRYLQEIFANEMAKLDDSFFQRLEESFLVEEDKKNERHPIFGNLADEVAYHRNYPTIYHLRKKLADSPEKADLRLIYLALAHIIKFRGHFLIEGKLNAENSDVAKLFYQLIQTYNQLFEESPLDEIEVDAKGILSARLSKSK**R**LEKLIAVFPNEKKNGLFGNIIALALGLTPNFKSNFDLTEDAKLQLSKDTYDDDLDELLGQIGDQYADLFSAAKNLSDAILLSDILRSNSEVTKAPLSASMVKRYDEHHQDLALLKTLVRQQFPEKYAEIFKDDTKNGYAGYVGIG**KKLRKRSGKL**ATQEEFYKFIKPILEKMDGAEELLAKL**N**RDDLLRKQRTFDNGSIPHQIHLKELHAILRRQEEFYPFLKENREKIEKILTFRIPYYVGPLARGNSRFAWLTRKSEEAITPWNFEEVVDKGASAQSFIERMTNFDEQLPNKKVLPKHSLLYEYFTVYNELTKVKYVTERMRKPEFLSGEQKKAIVDLLFKTNRKVTVKQLKEDYFKKIECFDSVEIIGVEDRFNASLGTYHDLLKIIKDKDFLDNEENEDILEDIVLTLTLFEDREMIEERLKTYAHLFDDKVMKQLKRRHYTGWGRLSRKMINGIRDKQSGKTILDFLKSDGFSNRNFMQLIHDDSLTFKEEIEKAQVSGQGDSLHEQIADLAGSPAIKKGILQTVKIVDELVKVMGHKPENIVIEMARENQTTTKGLQQSRERKKRIEEGIKELESQILKENPVENTQLQNEKLYLYYLQNGRDMYVDQELDINRLSDYDVDHIVPQSFIKDDSIDNKVLTRSVENRGKSDNVPSEEVVKKMKNYWRQLLNAKLITQRKFDNLTKAERGGLSEADKAGFIKRQLVETRQITKHVARILDSRMNTKRDKNDKPIREVKVITLKSKLVSDFRKDFQLYKVRDINNYHHAHDAYLNAVVGTALIKKYPKLESEFVYGDYKVYDVRKMIAKSEQEIGKATAKRFFYSNIMNFFKTEVKLANGEIRKRPLIETNGETGEVVWNKEKDFATVRKVLAMPQVNIVKKTEVQTGGFSKESILSKRESAKLIPRKKGWDTRKYGGFGSPTVAYSILVVAKVEKGKAKKLKSVKVLVGITIMEKGSYEKDPIGFLEAKGYKDIKKELIFKLPKYSLFELENGRRRMLASA**K**ELQKANELVLPQHLVRLLYYTQNISATTGSNNLGYIEQHREEFKEIFEKIIDFSEKYILKNKVNSNLKSSFDEQFAVSDSILLSNSFVSLLKYTSFGASGGFTFLDLDVKQGRLRYQTVTEVLDATLIYQSITGLYETRTDLSQLGGD

**ScRN++ (1375 aa)**

MEKKYSIGLDIGTNSVGWAVITDDYKVPSKKFKVLGNTNRKSIKKNLMGALLFDSGETAEATRLKRTARRRYTRRKNRIRYLQEIFANEMAKLDDSFFQRLEESFLVEEDKKNERHPIFGNLADEVAYHRNYPTIYHLRKKLADSPEKADLRLIYLALAHIIKFRGHFLIEGKLNAENSDVAKLFYQLIQTYNQLFEESPLDEIEVDAKGILSARLSKSK**K**LEKLIAVFPNEKKNGLFGNIIALALGLTPNFKSNFDLTEDAKLQLSKDTYDDDLDELLGQIGDQYADLFSAAKNLSDAILLSDILRSNSEVTKAPLSASMVKRYDEHHQDLALLKTLVRQQFPEKYAEIFKDDTKNGYAGYVGIG**KKLRKRSGKL**ATQEEFYKFIKPILEKMDGAEELLAKL**K**RDDLLRKQRTFDNGSIPHQIHLKELHAILRRQEEFYPFLKENREKIEKILTFRIPYYVGPLARGNSRFAWLTRKSEEAITPWNFEEVVDKGASAQSFIERMTNFDEQLPNKKVLPKHSLLYEYFTVYNELTKVKYVTERMRKPEFLSGEQKKAIVDLLFKTNRKVTVKQLKEDYFKKIECFDSVEIIGVEDRFNASLGTYHDLLKIIKDKDFLDNEENEDILEDIVLTLTLFEDREMIEERLKTYAHLFDDKVMKQLKRRHYTGWGRLSRKMINGIRDKQSGKTILDFLKSDGFSNRNFMQLIHDDSLTFKEEIEKAQVSGQGDSLHEQIADLAGSPAIKKGILQTVKIVDELVKVMGHKPENIVIEMARENQTTTKGLQQSRERKKRIEEGIKELESQILKENPVENTQLQNEKLYLYYLQNGRDMYVDQELDINRLSDYDVDHIVPQSFIKDDSIDNKVLTRSVENRGKSDNVPSEEVVKKMKNYWRQLLNAKLITQRKFDNLTKAERGGLSEADKAGFIKRQLVETRQITKHVARILDSRMNTKRDKNDKPIREVKVITLKSKLVSDFRKDFQLYKVRDINNYHHAHDAYLNAVVGTALIKKYPKLESEFVYGDYKVYDVRKMIAKSEQEIGKATAKRFFYSNIMNFFKTEVKLANGEIRKRPLIETNGETGEVVWNKEKDFATVRKVLAMPQVNIVKKTEVQTGGFSKESILSKRESAKLIPRKKGWDTRKYGGFGSPTVAYSILVVAKVEKGKAKKLKSVKVLVGITIMEKGSYEKDPIGFLEAKGYKDIKKELIFKLPKYSLFELENGRRRMLASA**K**ELQKANELVLPQHLVRLLYYTQNISATTGSNNLGYIEQHREEFKEIFEKIIDFSEKYILKNKVNSNLKSSFDEQFAVSDSILLSNSFVSLLKYTSFGASGGFTFLDLDVKQGRLRYQTVTEVLDATLIYQSITGLYETRTDLSQLGGD

**Spc+ (1365 aa)**

MEKKYSIGLDIGTNSVGWAVITDDYKVPSKKFKVLGNTNRKSIKKNLMGALLFDSGETAEATRLKRTARRRYTRRKNRIRYLQEIFANEMAKLDDSFFHRLEESFLVEEDKKHERHPIFGNIVDEVAYHEKYPTIYHLRKKLVDSTDKADLRLIYLALAHMIKFRGHFLIEGDLNPDNSDVDKLFIQLVQTYNQLFEENPINASGVDAKAILSARLSKSR**R**LENLIAQLPGEKKNGLFGNLIALSLGLTPNFKSNFDLAEDAKLQLSKDTYDDDLDNLLAQIGDQYADLFLAAKNLSDAILLSDILRVNTEITKAPLSASMIKRYDEHHQDLTLLKALVRQQLPEKYKEIFFDQSKNGYAGYIDGGASQEEFYKFIKPILEKMDGTEELLVKL**N**REDLLRKQRTFDNGSIPHQIHLGELHAILRRQEDFYPFLKDNREKIEKILTFRIPYYVGPLARGNSRFAWMTRKSEETITPWNFEEVVDKGASAQSFIERMTNFDKNLPNEKVLPKHSLLYEYFTVYNELTKVKYVTEGMRKPAFLSGEQKKAIVDLLFKTNRKVTVKQLKEDYFKKIECFDSVEISGVEDRFNASLGTYHDLLKIIKDKDFLDNEENEDILEDIVLTLTLFEDREMIEERLKTYAHLFDDKVMKQLKRRRYTGWGRLSRKLINGIRDKQSGKTILDFLKSDGFANRNFMQLIHDDSLTFKEDIQKAQVSGQGDSLHEQIADLAGSPAIKKGILQTVKIVDELVKVMGHKPENIVIEMARENQTTTKGLQQSRERKKRIEEGIKELESQILKENPVENTQLQNEKLYLYYLQNGRDMYVDQELDINRLSDYDVDHIVPQSFIKDDSIDNKVLTRSVENRGKSDNVPSEEVVKKMKNYWRQLLNAKLITQRKFDNLTKAERGGLSEADKAGFIKRQLVETRQITKHVARILDSRMNTKRDKNDKPIREVKVITLKSKLVSDFRKDFQLYKVRDINNYHHAHDAYLNAVVGTALIKKYPKLESEFVYGDYKVYDVRKMIAKSEQEIGKATAKRFFYSNIMNFFKTEVKLANGEIRKRPLIETNGETGEVVWNKEKDFATVRKVLAMPQVNIVKKTEVQTGGFSKESILSKRESAKLIPRKKGWDTRKYGGFGSPTVAYSILVVAKVEKGKAKKLKSVKVLVGITIMEKGSYEKDPIGFLEAKGYKDIKKELIFKLPKYSLFELENGRRRMLASA**K**ELQKANELVLPQHLVRLLYYTQNISATTGSNNLGYIEQHREEFKEIFEKIIDFSEKYILKNKVNSNLKSSFDEQFAVSDSILLSNSFVSLLKYTSFGASGGFTFLDLDVKQGRLRYQTVTEVLDATLIYQSITGLYETRTDLSQLGGD

**SpcRN+ (1365 aa)**

MEKKYSIGLDIGTNSVGWAVITDDYKVPSKKFKVLGNTNRKSIKKNLMGALLFDSGETAEATRLKRTARRRYTRRKNRIRYLQEIFANEMAKLDDSFFHRLEESFLVEEDKKHERHPIFGNIVDEVAYHEKYPTIYHLRKKLVDSTDKADLRLIYLALAHMIKFRGHFLIEGDLNPDNSDVDKLFIQLVQTYNQLFEENPINASGVDAKAILSARLSKSR**K**LENLIAQLPGEKKNGLFGNLIALSLGLTPNFKSNFDLAEDAKLQLSKDTYDDDLDNLLAQIGDQYADLFLAAKNLSDAILLSDILRVNTEITKAPLSASMIKRYDEHHQDLTLLKALVRQQLPEKYKEIFFDQSKNGYAGYIDGGASQEEFYKFIKPILEKMDGTEELLVKL**K**REDLLRKQRTFDNGSIPHQIHLGELHAILRRQEDFYPFLKDNREKIEKILTFRIPYYVGPLARGNSRFAWMTRKSEETITPWNFEEVVDKGASAQSFIERMTNFDKNLPNEKVLPKHSLLYEYFTVYNELTKVKYVTEGMRKPAFLSGEQKKAIVDLLFKTNRKVTVKQLKEDYFKKIECFDSVEISGVEDRFNASLGTYHDLLKIIKDKDFLDNEENEDILEDIVLTLTLFEDREMIEERLKTYAHLFDDKVMKQLKRRRYTGWGRLSRKLINGIRDKQSGKTILDFLKSDGFANRNFMQLIHDDSLTFKEDIQKAQVSGQGDSLHEQIADLAGSPAIKKGILQTVKIVDELVKVMGHKPENIVIEMARENQTTTKGLQQSRERKKRIEEGIKELESQILKENPVENTQLQNEKLYLYYLQNGRDMYVDQELDINRLSDYDVDHIVPQSFIKDDSIDNKVLTRSVENRGKSDNVPSEEVVKKMKNYWRQLLNAKLITQRKFDNLTKAERGGLSEADKAGFIKRQLVETRQITKHVARILDSRMNTKRDKNDKPIREVKVITLKSKLVSDFRKDFQLYKVRDINNYHHAHDAYLNAVVGTALIKKYPKLESEFVYGDYKVYDVRKMIAKSEQEIGKATAKRFFYSNIMNFFKTEVKLANGEIRKRPLIETNGETGEVVWNKEKDFATVRKVLAMPQVNIVKKTEVQTGGFSKESILSKRESAKLIPRKKGWDTRKYGGFGSPTVAYSILVVAKVEKGKAKKLKSVKVLVGITIMEKGSYEKDPIGFLEAKGYKDIKKELIFKLPKYSLFELENGRRRMLASA**K**ELQKANELVLPQHLVRLLYYTQNISATTGSNNLGYIEQHREEFKEIFEKIIDFSEKYILKNKVNSNLKSSFDEQFAVSDSILLSNSFVSLLKYTSFGASGGFTFLDLDVKQGRLRYQTVTEVLDATLIYQSITGLYETRTDLSQLGGD

**SpcRN**++ **(1375 aa)**

MEKKYSIGLDIGTNSVGWAVITDDYKVPSKKFKVLGNTNRKSIKKNLMGALLFDSGETAEATRLKRTARRRYTRRKNRIRYLQEIFANEMAKLDDSFFHRLEESFLVEEDKKHERHPIFGNIVDEVAYHEKYPTIYHLRKKLVDSTDKADLRLIYLALAHMIKFRGHFLIEGDLNPDNSDVDKLFIQLVQTYNQLFEENPINASGVDAKAILSARLSKSR**K**LENLIAQLPGEKKNGLFGNLIALSLGLTPNFKSNFDLAEDAKLQLSKDTYDDDLDNLLAQIGDQYADLFLAAKNLSDAILLSDILRVNTEITKAPLSASMIKRYDEHHQDLTLLKALVRQQLPEKYKEIFFDQSKNGYAGYIDGG**KKLRKRSGKL**ASQEEFYKFIKPILEKMDGTEELLVKL**K**REDLLRKQRTFDNGSIPHQIHLGELHAILRRQEDFYPFLKDNREKIEKILTFRIPYYVGPLARGNSRFAWMTRKSEETITPWNFEEVVDKGASAQSFIERMTNFDKNLPNEKVLPKHSLLYEYFTVYNELTKVKYVTEGMRKPAFLSGEQKKAIVDLLFKTNRKVTVKQLKEDYFKKIECFDSVEISGVEDRFNASLGTYHDLLKIIKDKDFLDNEENEDILEDIVLTLTLFEDREMIEERLKTYAHLFDDKVMKQLKRRRYTGWGRLSRKLINGIRDKQSGKTILDFLKSDGFANRNFMQLIHDDSLTFKEDIQKAQVSGQGDSLHEQIADLAGSPAIKKGILQTVKIVDELVKVMGHKPENIVIEMARENQTTTKGLQQSRERKKRIEEGIKELESQILKENPVENTQLQNEKLYLYYLQNGRDMYVDQELDINRLSDYDVDHIVPQSFIKDDSIDNKVLTRSVENRGKSDNVPSEEVVKKMKNYWRQLLNAKLITQRKFDNLTKAERGGLSEADKAGFIKRQLVETRQITKHVARILDSRMNTKRDKNDKPIREVKVITLKSKLVSDFRKDFQLYKVRDINNYHHAHDAYLNAVVGTALIKKYPKLESEFVYGDYKVYDVRKMIAKSEQEIGKATAKRFFYSNIMNFFKTEVKLANGEIRKRPLIETNGETGEVVWNKEKDFATVRKVLAMPQVNIVKKTEVQTGGFSKESILSKRESAKLIPRKKGWDTRKYGGFGSPTVAYSILVVAKVEKGKAKKLKSVKVLVGITIMEKGSYEKDPIGFLEAKGYKDIKKELIFKLPKYSLFELENGRRRMLASA**K**ELQKANELVLPQHLVRLLYYTQNISATTGSNNLGYIEQHREEFKEIFEKIIDFSEKYILKNKVNSNLKSSFDEQFAVSDSILLSNSFVSLLKYTSFGASGGFTFLDLDVKQGRLRYQTVTEVLDATLIYQSITGLYETRTDLSQLGGD
